# Human Serum Metabolites as Potential Mediators from Type 2 Diabetes and Obesity to COVID-19 Severity and Susceptibility: Evidence from Mendelian Randomization Study

**DOI:** 10.3390/metabo12070598

**Published:** 2022-06-27

**Authors:** Chuiguo Huang, Mai Shi, Hongjiang Wu, Andrea O. Y. Luk, Juliana C. N. Chan, Ronald C. W. Ma

**Affiliations:** 1 Department of Medicine and Therapeutics, Prince of Wales Hospital, The Chinese University of Hong Kong, Shatin, New Territories, Hong Kong 999077, China; huangcg0727@link.cuhk.edu.hk (C.H.); mshi@link.cuhk.edu.hk (M.S.); hongjiangwu@cuhk.edu.hk (H.W.); andrealuk@cuhk.edu.hk (A.O.Y.L.); jchan@cuhk.edu.hk (J.C.N.C.); 2 Hong Kong Institute of Diabetes and Obesity, The Chinese University of Hong Kong, Shatin, New Territories, Hong Kong 999077, China; 3 Li Ka Shing Institute of Health Sciences, The Chinese University of Hong Kong, Shatin, New Territories, Hong Kong 999077, China

**Keywords:** COVID-19, human serum metabolites, type 2 diabetes, obesity, Mendelian randomization, mediation analysis

## Abstract

Obesity, type 2 diabetes (T2D), and severe coronavirus disease 2019 (COVID-19) are closely associated. The aim of this study was to elucidate the casual and mediating relationships of human serum metabolites on the pathways from obesity/T2D to COVID-19 using Mendelian randomization (MR) techniques. We performed two-sample MR to study the causal effects of 309 metabolites on COVID-19 severity and susceptibility, based on summary statistics from genome-wide association studies (GWAS) of metabolites (*n* = 7824), COVID-19 phenotypes (*n* = 2,586,691), and obesity (*n* = 322,154)/T2D traits (*n* = 898,130). We conducted two-sample network MR analysis to determine the mediating metabolites on the causal path from obesity/T2D to COVID-19 phenotypes. We used multivariable MR analysis (MVMR) to discover causal metabolites independent of body mass index (BMI). Our MR analysis yielded four causal metabolites that increased the risk of severe COVID-19, including 2-stearoylglycerophosphocholine (OR 2.15; 95% CI 1.48–3.11), decanoylcarnitine (OR 1.32; 95% CI 1.17–1.50), thymol sulfate (OR 1.20; 95% CI 1.10–1.30), and bradykinin-des-arg(9) (OR 1.09; 95% CI 1.05–1.13). One significant mediator, gamma-glutamyltyrosine, lay on the causal path from T2D/obesity to severe COVID-19, with 16.67% (0.64%, 32.70%) and 6.32% (1.76%, 10.87%) increased risk, respectively, per one-standard deviation increment of genetically predicted T2D and BMI. Our comprehensive MR analyses identified credible causative metabolites, mediators of T2D and obesity, and obesity-independent causative metabolites for severe COVID-19. These biomarkers provide a novel basis for mechanistic studies for risk assessment, prognostication, and therapeutic purposes in COVID-19.

## 1. Introduction

Since the outbreak of the coronavirus disease 2019 (COVID-19) in late 2019, as of May 2022, more than 400 million people were infected, with 6 million deaths worldwide [1]. There is marked heterogeneity in the clinical course of COVID-19 due to severe acute respiratory syndrome coronavirus 2 (SARS-CoV-2), ranging from asymptomatic infection to acute respiratory failure or death [2]. There is global evidence indicating that severe COVID-19 is closely linked to preexisting conditions such as obesity, diabetes, hypertension, cardiovascular–renal disease, heart failure, chronic respiratory disease, and cancers [3,4,5,6,7,8].

Among these conditions, type 2 diabetes (T2D) [5,9] and obesity [3] are major risk factors for severe COVID-19 requiring hospitalization, assisted ventilation, and premature death. However, the underlying molecular pathways involved in the association between T2D/obesity and COVID-19 remain unclear [5]. Recent research has proposed metabolic biomarkers as functional intermediates to investigate the impact of genetics on metabolic disorders including T2D and obesity. Metabolites are intermediates or end products of metabolic pathways that may be perturbed in cardiometabolic diseases [10]. To this end, aberrant metabolites due to abnormal energy metabolism in diabetes and obesity may mediate the susceptibility and severity of COVID-19. Recently, metabolomic analysis in patients with COVID-19 identified serum metabolites and lipidomic markers related to disease severity [11,12]. However, the small sample size with insufficient statistical power might have led to over- or underestimating the effect of these biomarkers. Besides, the cross-sectional and observational nature of these studies precluded ascertainment of causal nature, in part because of unmeasured or unknown confounders.

Mendelian randomization (MR) is a statistical approach that uses randomly allocated genetic variants as instruments to verify the causal nature of modifiable exposures on clinical outcomes [13]. If designed properly, MR analysis can provide credible causal inference for biomarkers that may be prone to measurement error with small sample sizes or unknown confounders. Recent MR studies suggested that obesity, but not T2D or glycemic traits, was causally associated with severe COVID-19 [4,14]. Hence, to disentangle the causal relationship among obesity/T2D, metabolites, and COVID-19 susceptibility/severity, we adopted a network MR analysis framework using summary statistics from large genome-wide association studies (GWASs) in this study. We aimed to systematically evaluate the mediating roles of human serum metabolites in the link between obesity/T2D and COVID-19. To this end, we used genetic variants as instruments and performed relevant multivariable MR (MVMR) analysis to explain the potential pleiotropy when estimating the mediation effects of metabolites.

## 2. Results

### 2.1. Study Overview

**Figure 1:**
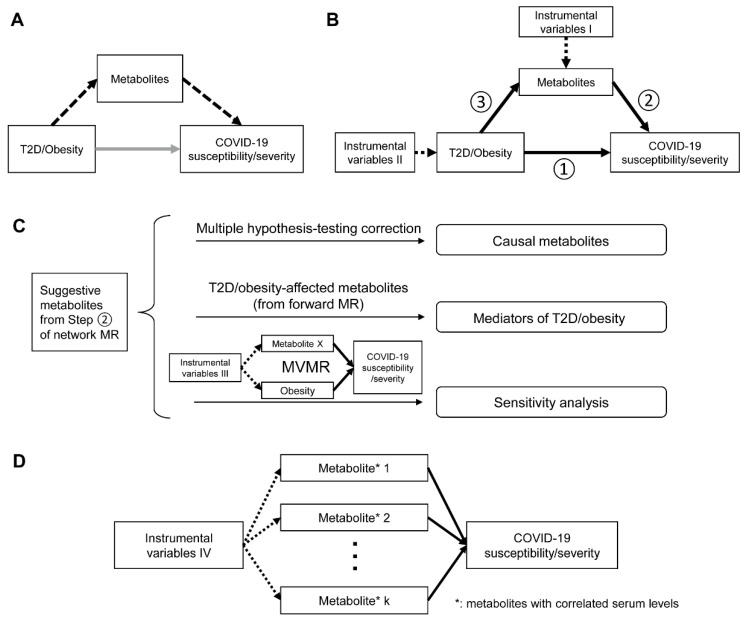
Overview of the study design. (**A**) Directed acyclic graphs illustrating the hypothetical causal model of T2D/obesity, metabolites, and COVID-19 susceptibility/severity. Dashed arrow: indirect effect via mediator; grey arrow: direct effect. (**B**) Framework of network Mendelian randomization. The analysis flow started from the estimation of total effect of T2D/obesity on the outcomes (①), followed by outcome-related metabolite screening (②). The final step in the network MR was an assessment of the causality between metabolites and T2D/obesity (③). Dashed arrow: instrumental variables of exposures; black arrow: directions of causal association. (**C**) Further analyses on the metabolites with suggestive associations with outcomes from network MR. These analyses included multiple hypothesis-testing corrections to prioritize credible causative metabolites, multivariable Mendelian randomization for single metabolites adjusting for BMI, and mediation analysis to identify the mediators. Dashed arrow: instrumental variables of exposures; black arrow: directions of causal association. (**D**) Multivariable Mendelian randomization for metabolites with correlated serum levels. Dashed arrow: instrumental variables of exposures; black arrow: directions of causal association.

The framework of MR analysis in this study is described by the directed acyclic graphs (DAGs) shown in Figure 1. We conducted both two-sample network MR and MVMR to test the hypothesis that the link between obesity/T2D and COVID-19 was mediated by metabolites (Figure 1A). The network MR included three steps when we performed the analysis using univariable two-sample MR approaches (Figure 1B). We first explored the total effect of T2D/obesity on the COVID-19 phenotypes as outcomes (step 1). We then investigated the causal relationship between human serum metabolites and outcomes to prioritize suggestive candidates (step 2). We finalized the network MR by assessing the causal relationships between these COVID-19-associated metabolites and T2D/obesity (step 3). Mediation effects of the metabolites from T2D/obesity to COVID-19 outcomes were derived from the estimates obtained in the network MR. To further address the potential pleiotropic effects, we supplemented the mediation analysis using MVMR to estimate the residual effects of each metabolite on the outcomes, adjusted for the genetic instruments of T2D/obesity (Figure 1C). Finally, to account for confounding due to correlations among metabolites within the same biochemical categories or pathways, we consolidated our findings by performing another MVMR analysis for the correlated metabolites (Figure 1D). And Supplementary Material Table S1 summarizes the detailed information from the GWAS summary data used in this study.

### 2.2. Instrument Strength

The number of genetic variants used as instrumental variables of the 309 human serum metabolites under investigation varied from 3 to 284, with a median number of 15. These instruments explained 0.9–87.6% of the variance for their respective metabolites. We discarded seven metabolites from our MR analysis because of their aberrant R^2^ resulting from the small effective sample size. The minimum F-statistic of the remaining 302 metabolites was 21.53. All these genetic instrumental variables were sufficiently informative (F > 10) for network MR analysis (Supplementary Material Table S2).

### 2.3. Network MR Step 1: Causal Associations between T2D, Glycemic Traits, Adiposity Traits and COVID-19 Phenotypes

Using the latest version of COVID-19 GWAS data, we confirmed findings from previous MR studies on the causal relationships between T2D, glycemic traits, adiposity traits and COVID-19 phenotypes (Supplementary Material Figure S1 and Supplementary Material Table S3a). There were suggestive associations between T2D and COVID-19 severity (B2: OR_IVW_ 1.05; 95% CI 1.01–1.10; P_IVW_ = 0.0067) and susceptibility (C2: OR_IVW_ 1.02; 95% CI 1.00–1.04; P_IVW_ = 0.039), albeit with possible horizontal pleiotropy (P_MR–PRESSO Global_ < 0.05, P_MR–Egger intercept_ < 0.05, Supplementary Material Table S3b). After adjusting for BMI (T2DadjBMI), we found that all causal associations between T2D and COVID-19 phenotypes were rendered nonsignificant (P_IVW_ > 0.05). In addition, BMI but not glycemic traits (FG, FI, HbA1c, and 2hGlu) was causally associated with all three COVID-19 phenotypes (Supplementary Material Table S3a; A2: OR_IVW_ 1.70; 95% CI 1.39–2.07; P_IVW_ < 0.001; B2: OR_IVW_ 1.52; 95% CI 1.33–1.75; P_IVW_ < 0.001; C2: OR_IVW_ 1.14; 95% CI 1.08–1.20; P_IVW_ < 0.001). The causal associations between BMI and COVID-19 severity (B2) and susceptibility (C2) remained robust in sensitivity analyses and passed the tests of pleiotropic effects (Supplementary Material Table S3a,b). No causal relationship was observed between the outcomes and another adiposity trait, WHR, either with or without BMI adjustment.

### 2.4. Network MR Step 2: Causal Association between Human Serum Metabolites and COVID-19 Phenotypes

**Figure 2:**
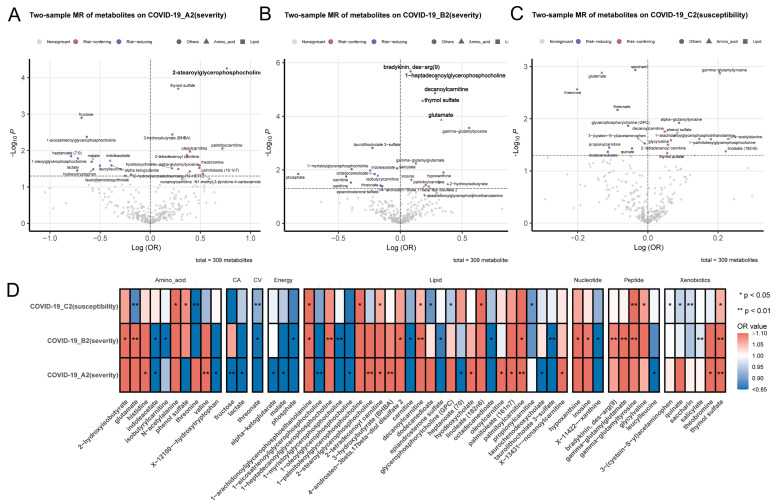
Causal associations between human serum metabolites and COVID-19 phenotypes. (**A**–**C**) Volcano plots that include both odds ratios (OR) in log scale and P-values estimated by the inverse-variance weighted method (multiplicative random-effects model) for each COVID-19 phenotype. Six metabolites that passed the Bonferroni correction (P_IVW_ < 0.05/302 = 1.66 × 10^−4^) are shown in boldface type. (**D**) Heatmap of the 56 metabolites that showed causal association with at least one COVID-19 phenotype at nominal significance (P_IVW_ < 0.05). CA: carbohydrate; CV: cofactors and vitamins.

From the 302 metabolites with valid IVs, initial screening yielded 70 suggestive associations with COVID-19 phenotypes at nominal significance levels (P_IVW_ < 0.05). After removing associations with potential horizontal pleiotropic effects (P_MR–Egger intercept_ < 0.05), we obtained 56 unique metabolites that contributed to 24, 24, and 18 causal associations with COVID-19 phenotypes A2 (severity), B2 (severity), and C2 (susceptibility), respectively (Figure 2 and Supplementary Material Table S4). These metabolites were enriched in pathways implicated in “carnitine metabolism” (A2; hypergeometric test P_hyper_ = 0.013), “Krebs cycle” (A2; P_hyper_ = 0.032), “purine metabolism” (B2; P_hyper_ = 0.0016), and “food component, plant” (C2; P_hyper_ = 0.013). Six causal associations between metabolites and COVID-19 severity remained significant per 1 log10 unit increase in the serum level after correction for multiple hypothesis testing using the conservative Bonferroni threshold (P_IVW_ < 0.05/302 = 1.66 × 10^−4^): 2-stearoylglycerophosphocholine with A2 (odds ratio from IVW method (OR_IVW_) 2.15; 95% CI 1.48–3.11; P_IVW_ = 5.54 × 10^−5^), bradykinin-des-arg(9) with B2 (OR_IVW_ 1.09; 95% CI 1.05–1.13; P_IVW_ = 2.11 × 10^−6^), 1-heptadecanoylglycerophosphocholine with B2 (OR_IVW_ 1.34; 95% CI 1.18–1.52; P_IVW_ = 4.04 × 10^−6^), decanoylcarnitine with B2 (OR_IVW_ 1.32; 95% CI 1.17–1.50; P_IVW_ = 1.36 × 10^−5^), thymol sulfate with B2 (OR_IVW_ 1.20; 95% CI 1.10–1.30; P_IVW_ = 2.66 × 10^−5^), and glutamate with B2 (OR_IVW_ 1.39; 95% CI 1.17–1.65; P_IVW_ = 1.36 × 10^−4^) (Supplementary Material Figure S2). These six metabolites were involved in “lysolipid”, “polypeptide”, “carnitine metabolism” and “glutamate metabolism” pathways. None of the six had pairwise serum-level correlation larger than 0.04 (|r| > 0.2). The causal associations of 2-stearoylglycerophosphocholine, bradykinin-des-arg(9), decanoylcarnitine, and thymol sulfate with COVID-19 severity remained robust in sensitivity analyses (P_weighted-median_ < 0.05; Table 1 and Supplementary Material Table S4).

### 2.5. Network MR Step 3: Causal Relationship between COVID-19-Related Metabolites and T2D/Obesity

We next evaluated the relationship between T2D/obesity and each of the 56 metabolites from our screening above by treating genetically predicted T2D or BMI as exposure in the two-sample MR analysis. We identified eight metabolites causally affected by the genetic predisposition to T2D per unit increase in log odds of T2D risk (Supplementary Material Table S5): inosine (β_IVW_ = −0.054; 95% CI (−0.095, −0.012); P_IVW_ =1.00 × 10^−2^), heptanoate (β_IVW_ = −0.014; 95% CI (−0.023, −0.005); P_IVW_ = 3.20 × 10^−3^), valine (β_IVW_ = 0.006; 95% CI (0.001, 0.012); P_IVW_ =1.69 × 10^−2^), lactate (β_IVW_ = 0.011; 95% CI (0.001, 0.021); P_IVW_ =3.10 × 10^−2^), indoleacetate (β_IVW_ = 0.016; 95% CI (0.001, 0.030); P_IVW_ = 3.52 × 10^−2^), gamma-glutamyltyrosine (β_IVW_ = 0.016; 95% CI (0.007, 0.025); P_IVW_ = 4.73 × 10^−4^), glutamate (β_IVW_ = 0.027; 95% CI (0.013, 0.041); P_IVW_ = 1.83 × 10^−4^), and fructose (β_IVW_ = 0.023; 95% CI (0.007, 0.038); P_IVW_ = 3.63 × 10^−3^). In MVMR analysis, in which the potential confounding effect of T2D on the metabolite-COVID associations was adjusted, two out of the eight metabolites had their causal associations with the outcomes remain at nominal significance level (Supplementary Material Figure S3 and Supplementary Material Table S6): gamma-glutamyltyrosine with B2 (OR_MV-IVW_: 2.15, 95% CI 1.27–4.09; P_MV-IVW_ = 2.02 × 10^−2^) and glutamate with C2 (OR_MV-IVW_: 0.73, 95% CI 0.56–0.96; P_MV-IVW_ = 2.46 × 10^−2^).

We also identified 14 metabolites which were causally affected by BMI (P_IVW_ < 0.05; Supplementary Material Table S7). Of these, six were inversely correlated with BMI: phenol sulfate (β_IVW_ = −0.060; 95% CI [−0.110, −0.011]; P_IVW_ = 0.0169), heptanoate (β_IVW_ = −0.031; 95% CI [−0.049, −0.013]; P_IVW_ = 0.0006), 1-oleoylglycerophosphocholine (β_IVW_ = −0.031; 95% CI [−0.056, −0.006]; P_IVW_ = 0.0144), 2-stearoylglycerophosphocholine (β_IVW_ = −0.038; 95% CI [−0.071, −0.004]; P_IVW_ = 0.0275), and 2-tetradecenoyl carnitine (β_IVW_ = −0.046; 95% CI [−0.091, −0.002]; P_IVW_ = 0.042). The remaining eight had incremental serum levels ranging from 0.014 (valine) to 0.123 log10 unit (quinate) per 1 SD increase in BMI. In MVMR analysis, five metabolites retained their causal associations with the outcomes at P_MV-IVW_ < 0.05 (Supplementary Material Figure S4 and Supplementary Material Table S8): valine with A2 (OR_MV-IVW_ 8.89; 95% CI 1.22–64.64; P_MV-IVW_ = 3.09 × 10^−2^), alpha-glutamyltyrosine with both A2 (OR_MV-IVW_ 1.40; 95% CI 1.02–1.94; P_MV-IVW_ = 3.92 × 10^−2^) and C2 (OR_MV-IVW_ 1.10; 95% CI 1.02–1.19; P_MV-IVW_ = 1.24 × 10^−2^), gamma-glutamyltyrosine with B2 (OR_MV-IVW_ 1.62; 95% CI 1.03–2.54; P_MV-IVW_ = 3.49 × 10^−2^), and glutamate with C2 (OR_MV-IVW_ 0.82; 95% CI 0.69–0.98; P_MV-IVW_ = 3.07 × 10^−2^).

### 2.6. Mediation Effects of Metabolites

**Figure 3:**
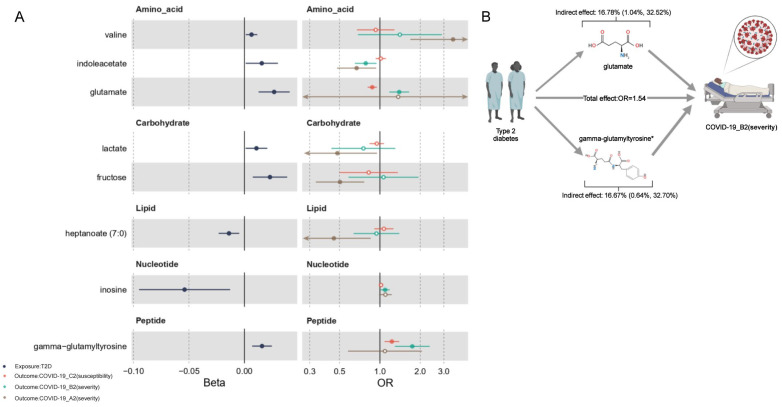
(**A**) Forest plots of 8 T2D-affected human serum metabolites. Left panel: causal effects of genetically predicted BMI on the serum levels of metabolites. Beta (x-axis) was the log10 unit changes of metabolite level per one-standard-deviation (1 SD) increase in T2D (T2D as exposure). Right panel: causal effects of the 8 T2D-affected metabolites on the COVID-19 phenotypes. OR (x-axis) was the estimated odds ratio of the outcome per 1 log10 unit increase in the serum levels of metabolites. Solid dots indicate significant associations (P_IVW_ < 0.05). (**B**) Diagrams illustrating the mediation effects of mediators on the causal path from T2D to COVID-19 phenotypes A2 (red), B2 (red), and C2 (yellow). *: Indicator of statistical significance of multivariable Mendelian randomization (MVMR) analysis for causal associations between mediators and COVID-19 phenotypes conditioning on genetically predicted T2D risk.

We assessed the mediating effects of metabolites that were associated with both exposures (T2D/BMI) and outcomes (COVID-19 phenotypes) in the network MR analysis. For T2D, we detected two significant positive indirect effects (Table 2 and Figure 3): glutamate with B2 (indirect effect in log OR scale (Beta_indirect_) 0.009 per unit increase in log odds of T2D risk; 95% CI [0.002, 0.015]) and gamma-glutamyltyrosine with B2 (Beta_indirect_ 0.009; 95% CI [0.002, 0.016]). The proportion of the association between T2D and COVID-19 phenotypes mediated by these metabolites were 16.78% (95% CI [1.04%, 32.52%]) and 16.67% (95% CI [0.64%, 32.70%]), respectively. In combination with results from MVMR analysis, we considered gamma-glutamyltyrosine as a mediator with strong evidence in the association between T2D and COVID-19 severity. Meanwhile, glutamate was a mediator with moderate evidence for both COVID-19 severity and susceptibility.

**Figure 4:**
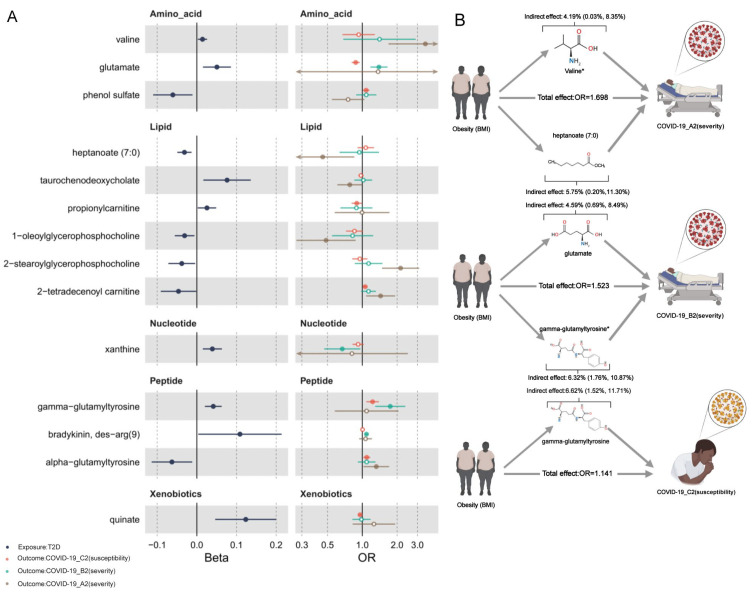
(**A**) Forest plots of 14 BMI-affected human serum metabolites. Left panel: causal effects of genetically predicted BMI on the serum levels of metabolites. Beta (x-axis) was the log10 unit changes of metabolite level per one-standard-deviation (1 SD) increase in BMI (BMI as exposure). Right panel: causal effects of the 14 BMI-affected metabolites on the COVID-19 phenotypes. OR (x-axis) was the estimated odds ratio of the outcome per 1 log10 unit increase in the serum levels of metabolites. Solid dots indicate significant associations (P_IVW_ < 0.05). (**B**) Diagrams illustrating the mediation effects of mediators on the causal path from obesity to COVID-19 phenotypes A2, B2 and C2. *: Indicator of statistical significance of multivariable Mendelian randomization (MVMR) analysis for casual associations between mediators and COVID-19 phenotypes conditioning on BMI.

Among the 14 BMI-associated metabolites, we observed 5 statistically significant positive-mediating effects on the causal path from BMI to COVID-19 phenotypes (Table 3 and Figure 4): valine with A2 (indirect effect in log OR scale (Beta_indirect_) 0.018 per 1 SD increase in BMI; 95% CI [0.001, 0.036]), heptanoate (7:0) with A2 (Beta_indirect_ 0.025; 95% CI [0.001, 0.049]), glutamate with B2 (Beta_indirect_ 0.017; 95% CI [0.002, 0.031]), and gamma-glutamyltyrosine with both B2 and C2 (Beta_indirect_ 0.023; 95% CI [0.006, 0.04]; Beta_indirect_ 0.009; 95% CI [0.002, 0.015]). The proportions mediated were 4.19% (95% CI [0.03%, 8.35%]), 5.75% (95% CI [0.20%, 11.30%]), 4.59% (95% CI [0.69%, 8.49%]), 6.32% (95% CI [1.76%, 10.87%]), and 6.62% (95% CI [1.52%, 11.71%]), respectively. MVMR analysis supported that valine and gamma-glutamyltyrosine were two mediators with sufficient evidence in the association between obesity and COVID-19 severity.

### 2.7. Reassessment on the Independent Causal Effects of Metabolites and COVID-19 Phenotypes

Finally, to adjust for close correlations among metabolites, we reassessed the direct effects of causal and mediating metabolites on COVID-19 phenotypes by conditioning on their corresponding correlated metabolites. None of the causal associations with the outcomes detected above arose from correlation with other metabolites except for that of glutamate (Supplementary Material Table S9). The causal relationship between glutamate and COVID-19 phenotypes was negated after adjustment of its correlated metabolite gamma-glutamyltyrosine.

## 3. Discussion

Obesity, diabetes, and COVID-19 are closely associated, although the nature of these correlations remains uncertain. In this study, we performed comprehensive MR analyses to find genetic evidence helpful for resolving the interrelationships between human serum metabolites, obesity/T2D, and COVID-19. Using genetic variants as instruments, we prioritized 56 human serum metabolites with possible causal associations with COVID-19 severity or susceptibility. From these candidates, we discovered (i) metabolites causally increasing the risk of COVID-19 severity and (ii) metabolites exhibiting mediating effects on the pathway from T2D/obesity to COVID-19. These findings underscored the importance of dysregulation of lipid, choline, and carnitine metabolism, as well as that of inflammatory processes, in severe COVID-19.

From the metabolites found to increase the risk of severe COVID-19, we pinpointed key contributors in the pathways related to COVID-19 progression. These included 2-stearoylglycerophosphocholine (lysolipid metabolism), bradykinin-des-arg(9) (inflammation), decanoylcarnitine (carnitine metabolism), and thymol sulfate (dietary plant phenol). In a study of infants hospitalized for bronchiolitis, researchers reported an association between respiratory viruses and glycerophosphocholines (GCP), the metabolites from which 2-stearoylglycerophosphocholine is derived. This finding supported the role of GCP-related lipid metabolism in virus infection [15]. Bradykinin-des-arg(9), an active metabolite of bradykinin, is known to have a higher rate of degradation in women than in men [16]. The risk-conferring role of bradykinin-des-arg(9) in our analysis might explain the worse prognosis among men infected with COVID-19 [17]. Decanoylcarnitine is involved in carnitine and fatty acid metabolism, both of which are implicated in energy production. Carnitine deficiency and dysregulation have been reported in endocrine disorders such as diabetes [18] and may therefore contribute to the link between T2D and COVID-19 risk. Thymol sulfate is the metabolized form of thymol in human plasma. Thymol is a naturally occurring plant phenol with antibiotic and antiinflammatory properties [19]. The positive causal association between thymol sulfate and COVID-19 severity implied a relationship between host thymol metabolism and COVID-19 risk, suggesting the potential therapeutic use of thymol for disease prevention.

We further expanded our understanding of the aforementioned 56 candidates by interrogating their causal and mediating relationships with T2D and obesity, two well-reported risk factors for COVID-19 from observational studies. We found sufficient evidence to support the role of serum gamma-glutamyltyrosine in mediating the causal relationship between genetically predicted T2D risk and COVID-19 severity. Gamma-glutamyltyrosine is involved in the gamma-glutamyl amino acid pathway, which is closely related to insulin sensitivity [20]. Plasma gamma-glutamyltyrosine was reported to be positively correlated with HOMA-IR (homeostasis model assessment-estimated insulin resistance) [21], and downregulated plasma levels of this metabolite were observed in T2D patients who were on metformin treatment [22]. The mediation analysis results thus implicated a plausible mechanism underlying the increased risk of severe COVID-19 in the T2D population. In our estimation of the proportion mediated, we found that serum gamma-glutamyltyrosine accounted for over 16% of the association between T2D and COVID-19 severity. Although the proportion estimate was statistically significant, we would interpret this result cautiously, given the weak total effect of T2D on COVID-19 severity indicated in step 1 of our network MR analysis.

The positive mediating effect of serum gamma-glutamyltyrosine in the associations between obesity and COVID-19 severity/susceptibility further confirmed the critical function of this metabolite in linking metabolic disorders and COVID-19. Besides this, we synthesized strong evidence for another mediator, valine, that contributed to the increased risk of severe COVID-19 caused by obesity. Valine is a branched-chain amino acid (BCAA). Elevated circulating levels of BCAAs, frequently observed in individuals with obesity, have been reported to be associated with worse metabolic health and increased risk of insulin resistance followed by the development of T2D [21,22,23]. The causal relationship between valine and COVID-19 severity thus suggested BCAA metabolism as a potential therapeutic pathway for alleviating the disease progression in infected patients.

Moreover, we observed metabolites with negative mediating effects that might neutralize the mediators with positive effects. For example, genetically predicted T2D causally increased the serum fructose level, but serum fructose was found to reduce the risk of COVID-19 severity in our study. Since no inverse correlation among metabolites with opposite mediating effects was observed, these findings probably explained the discrepancy between observational studies and MR studies on the relationship between T2D and COVID-19 phenotypes.

We conducted MVMR analysis for all significant findings presented in the network MR to examine the residual associations of metabolites with COVID-19 phenotypes conditioning on their correlated counterparts. In each analysis, we included only a pair of correlated metabolites in the MVMR model, since too many exposures would reduce the power of the model [24]. Except for glutamate, most of the causal associations remained significant after adjusting for intercorrelations. The associations between glutamate and COVID-19 B2 and C2 phenotypes were attenuated after adjusting for its correlated metabolites gamma-glutamyltyrosine (r = 0.35) and gamma-glutamylglutamate (r = 0.61). There are three possible explanations for this. First, glutamate may be implicated in COVID-19 without being the key causal factor. Second, in our network MR analysis, glutamate was positively associated with COVID-19 severity but inversely associated with disease susceptibility, suggesting a nonlinear relationship between serum glutamate and COVID-19. Third, the conditional F-statistic in this MVMR analysis was small for glutamate (less than 10), suggesting possible bias due to weak instruments. That said, a previous report on the association between plasma glutamate level and incident cardiovascular events [25] suggests that the causal and mediating role of glutamate in COVID-19 warrants further investigation.

Our study had multiple strengths. This was the first attempt to use a comprehensive MR framework to dissect the interrelationships between metabolites, obesity/T2D, and COVID-19. The instruments for serum metabolite levels were derived from healthy Europeans, while the GWAS data of metabolic traits and COVID-19 phenotypes were generated from independent cohorts with few overlapping samples. The use of a large sample size for the different traits enabled our MR analysis to overcome limitations due to measured or unmeasured confounders between exposures and outcomes that cannot be addressed by a single observational study. We examined the instrument strength for each exposure in our two-sample network MR and evaluated the conditional instrument strength in the MVMR analysis. We also minimized potential biases due to horizontal pleiotropy, heterogeneity, and existence of outliers throughout our MR analyses. We applied stringent criteria in prioritizing credible causal associations, as we required the metabolites to be statistically significant in both primary and sensitivity analyses. Meanwhile, we also retained metabolites with weaker evidence and proposed them as potential candidates for future studies. Finally, we consolidated our findings in light of potential correlation among serum metabolites.

Our study also had limitations. First, we did not consider nonlinearity between metabolites and outcomes. A nonlinear MR analysis requires individual-level data and is usually implemented in one-sample design [26]. We used a two-sample design to leverage the availability of multiple large databases in order to increase statistical power and minimize confounding effects to draw more generalizable conclusions. Second, we did not assess trait–metabolite or metabolite–metabolite interactions. All MR analyses were conducted based on the assumption of no interaction between exposures and mediators (network MR) or exposures and exposures (MVMR). We assumed that the effects of exposures on both mediators and outcomes, as well as those of mediators on outcomes, were homogeneous. This assumption of homogeneity might not have fully addressed our research question, although MR methods that can account for interactions in two-sample design are still being developed. Last, we were not able to verify our findings using an independent metabolomic GWAS data because of a lack of similar datasets.

In summary, our comprehensive MR analyses identified human serum metabolites causally associated with COVID-19 susceptibility and severity. We found genetic evidence supporting gamma-glutamyltyrosine as a mediator on the causal path from T2D/obesity to COVID-19 severity. Our findings provide a landscape view of how circulating metabolites may affect COVID-19 progression. The proposed causal metabolites have the potential to be developed into clinical biomarkers for risk stratification and treatment assignment while providing the basis for mechanistic studies to unravel the pathophysiology of COVID-19 progression.

## 4. Materials and Methods

### 4.1. Data Sources

#### 4.1.1. Outcomes

We obtained the latest GWAS summary statistics for COVID-19 phenotypes from the COVID-19 Host Genetics Initiative (HGI: https://www.covid19hg.org/, accessed on 30 December 2021, round 6, released on 15 June 2021). The primary COVID-19 outcomes in our study included severe respiratory diseases (phenotype A2 defined in COVID-19 HGI) and hospitalization (phenotype B2) as indicators of severity and general COVID-19 (phenotype C2) as the indicator of disease susceptibility.

#### 4.1.2. Exposure and Mediators

We obtained the full GWAS summary statistics of human blood metabolites from comprehensive genetic association scan for more than 400 metabolites in 7824 adults from 2 European population studies: Cooperative Health Research in the Region of Augsburg (KORA; *n* = 1768) and TwinsUK (*n* = 6056), as reported by Shin et al. [27]. The metabolites were measured in either plasma or serum collected after overnight fasting in healthy individuals. Based on their chemical identity, these metabolites were classified into nine categories, including eight broad metabolic groups and an “unknown” group. The eight metabolic groups were amino acids, carbohydrates, cofactors and vitamins, energy, lipids, nucleotides, peptides, and xenobiotic metabolism. After stringent quality controls on the metabolomic data, the authors retained 309 known and 177 unknown metabolites for genetic analysis. In our study, we adopted the genetic associations, group/pathway information, and pairwise correlations of the 309 known metabolites for our MR analyses.

Genetic data were curated from the GWAS summary statistics for T2D with or without body mass index (BMI) adjustment from the DIAbetes Genetics Replication And Meta-analysis (DIAGRAM) Consortium [28]. GWAS summary data of other glycemic traits, including 2 h plasma glucose after a 75 g oral glucose tolerance test (2hGlu), fasting glucose (FG), fasting insulin (FI), and glycated hemoglobin (HbA1c), were curated from the Meta-Analyses of Glucose and Insulin-related traits Consortium (MAGIC), one of the largest genetic consortia on the glycemic traits of individuals without diabetes [29]. GWAS summary data for adiposity traits, including BMI and waist-to-hip ratio (WHR) with or without BMI adjustment, were curated from the Genetic Investigation of Anthropometric Traits (GIANT) Consortium [30]. For all the exposure data we adopted, we focused on those derived from European populations, since the majority of individuals included in the COVID-19 GWAS were of European decent. Supplementary Material Table S1 summarizes the detailed information from the GWAS database used in this study. All studies Were approved by a relevant ethical review board with participants’ informed consent.

### 4.2. Genetic Instruments

We employed a conventional threshold of genome-wide significance (*p* < 5 × 10^−8^) to select the candidates of genetic instruments for T2D, glycemic traits, and adiposity traits. A less stringent threshold (*p* < 1 × 10^−5^) was used for each of the 309 serum metabolites so as to explain a larger variation, since only a few genome-wide significant SNPs were identified. We performed a clumping procedure to prune the set of significant SNPs by setting a linkage disequilibrium threshold of r^2^ < 0.001 based on the reference panel of the European population from the 1000 Genome Project. We retained the independent SNPs with the lowest *p*-values within a 10,000 kb window. These SNPs were then harmonized with the outcome GWAS summary statistics; SNPs were discarded if they were either palindromic or not available in the outcome GWAS. We further applied the Steiger filtering method to avoid reverse causation in subsequent analyses by removing SNPs of which the association with the outcome was significantly stronger than that with the exposure [31]. The remaining SNPs were used as the final instrumental variables (IVs) for each trait and metabolite. To validate the instrument strength assumption for MR analysis, we estimated the proportion of variance in the exposure explained by genetic variants (*R*^2^) using the formula $R^{2}\;\approx \;\sum_{i}^{K}\frac{\beta_{i}^{2}}{\beta_{i}^{2}+N{\left(se\left(\beta_{i}\right)\right)}^{2}}$, where *β_i_* is the effect size of genetic instrument variant *i*, *N* is the effective sample size, *se*(*β_i_*) is the standard error of effect size for the genetic variant *i*, and *K* is the number of independent genetic variants [32]. We then calculated the F-statistic by $F=\frac{\left(N-K-1\right)R^{2}}{K\left(1-R^{2}\right)}$ and compared it with the empirical threshold of 10 to evaluate the strength of these genetic instruments.

In MVMR analysis, genetic instruments were first selected using the same thresholds as above for each trait or metabolite. We then pruned the composite instruments of multiple exposures under the MVMR investigation with the aforementioned parameters and reference panel. Only the independent SNPs selected in the analyses of all exposures were subject to harmonization with the outcome GWAS data followed by Steiger filtering. The remaining SNPs were considered as the genetic instruments for MVMR analysis. The instrument strength for MVMR analysis was evaluated using the conditional F-statistic [33].

### 4.3. Statistical Analysis

#### 4.3.1. Two-Sample Mendelian Randomization

We used the inverse-variance weighted (IVW) method with multiplicative random effects as the primary approach to estimate the causal effect of exposures on outcomes throughout our two-sample network MR analyses. We additionally performed sensitivity tests to assess the robustness of the results from primary analyses under different assumptions about the instrument validity and pleiotropic effects, using the weighted median method and the MR–Egger method. The two methods rely on weaker assumptions than the IVW method and can provide reliable effect estimates when there exist invalid genetic instruments or horizontal pleiotropy [34]. The weighted median method requires at least half of IVs to be valid, thus being robust to outliers in instruments. The MR–Egger method builds a weighted regression of IV-outcome associations on IV-exposure associations, with an intercept term representing the average pleiotropic effect. We considered the presence of directional pleiotropy if the intercept estimate of the MR–Egger regression significantly deviated from 0 (P_MR–Egger intercept_ < 0.05). We then adopted the regression slope as the pleiotropy-corrected estimate of the causal effect. Furthermore, we evaluated the heterogeneity between variant-specific causal estimates using MR–Pleiotropy Residual Sum and Outlier (MR–PRESSO) global test and Cochran’s Q statistic. When substantial heterogeneity was observed (P_MR–PRESSO Global_ < 0.05), we applied the MR–PRESSO outlier test to obtain the pleiotropy-corrected IVW estimates with heterogeneous outliers removed. We also classified the metabolites into two evidence levels based on their robustness in primary and sensitivity analyses. Metabolites with significant associations in both were considered as credible candidates, while those with significance only in primary analysis were considered to have suggestive evidence for the causal relationship. All the two-sample MR analyses were performed in R (version 4.1.2) using the R packages *TwoSampleMR* (version 0.5.6) and *MRPRESSO* (version 1.0) [31,35,36].

#### 4.3.2. Pathway Analysis

We followed the pathway annotation of the 309 metabolites in the original study by Shin et al. [27] to conduct pathway enrichment analysis for the causal metabolites of COVID-19 phenotypes identified in our network MR analysis. We adopted a hypergeometric test to assess the overrepresentation of pathways enriched in the prioritized metabolites, using all 309 metabolites as the background. A hypergeometric test *p*-value (P_hyper_) less than 0.05 was considered statistically significant in the enrichment analysis.

#### 4.3.3. Mediation Analysis

Serum metabolites with causal associations detected in both steps 2 and 3 of the network MR were regarded as potential mediators on the causal paths from obesity/T2D to COVID-19 phenotypes. We assessed their mediating effects by multiplying the two causal effect estimates from network MR (i.e.,$\;{\hat{\beta }}_{exposure\to mediator}\times {\hat{\beta }}_{mediator\to outcome}$, the product of coefficients method in conventional mediation analysis) [24]. For each causal metabolite, the product provided an estimate of the indirect effect from exposure to outcome mediated by the serum level of this particular metabolite (mediation effect). We also computed the proportion of the effect explained by the mediating metabolite by dividing the aforementioned product by the total effect: $\;\frac{{\hat{\beta }}_{exposure\to mediator}\times {\hat{\beta }}_{mediator\to outcome}}{{\hat{\beta }}_{exposure\to outcome}}\times 100\%$. The denominator was estimated by the IVW method (or the outlier-corrected MR–PRESSO method when applicable) in step 1 of the network MR. This proportion provided another measure of mediation expressed as the contribution of the metabolite in the causal association between exposure and outcome. We used the multivariate delta method [37,38] to calculate the confidence intervals for the indirect effects and proportions mediated. Metabolites with mediation effects significantly different from 0 were considered as the mediators in the pathway from obesity/T2D to COVID-19.

#### 4.3.4. Multivariable Mendelian Randomization (MVMR)

Multivariable MR analysis is an extension of univariable MR analysis for quantifying the direct effects of multiple exposures on the outcome [24]. MVMR analysis estimates the independent causal relationship between exposure and the outcome conditioning on the effects of other exposures. The multiple exposures could be either confounders, mediators, or colliders of each other or related to pleiotropic pathways. To test the robustness of the causal association between metabolites and COVID-19 phenotypes in our primary analysis, we performed sensitivity analyses by constructing an MVMR model that included obesity/T2D as the covariable for each suggestive metabolite from step 2 of the network MR. This MVMR model provided estimates for the direct causal effects of the metabolite on outcomes independently of obesity/T2D, thus adjusting for possible pleiotropic effects shared by exposures (obesity/T2D) and mediators (metabolites).

On the other hand, since metabolites with correlated serum levels were likely to have shared genetic factors, it was difficult to find specific genetic instruments for each of the correlated metabolites without loss of statistical power. Two-sample MR based on nonunique IVs has limited power to distinguish true causation from correlation with the causal metabolites. We therefore performed another MVMR analysis targeting at the correlated metabolites. From the supplementary information from the study by Shin et al., we identified metabolite pairs with serum-level correlation larger than 0.2 (|r| > 0.2, where r was the Pearson’s correlation coefficient) as correlated metabolites. We adopted the multivariable extension of the IVW method for all the MVMR analyses in this study, using the R packages MVMR (version 0.3) [33] and MendelianRandomization (version 0.5.1) [39].

Based on the MVMR results, we assigned different evidence levels to the potential mediators. Metabolites that both remained causally associated with the outcomes in MVMR and had statistically significant positive-mediating effects were considered to be mediators with strong evidence. Meanwhile, metabolites that met only one of the two criteria were regarded as mediators with moderate evidence. The remaining metabolites that failed both were proposed as potential mediators.

## Figures and Tables

**Table 1:** Summary of the primary findings in this study.

Methods	Role of Metabolites	Directed Acyclic Graph	Evidence Level	COVID-19 A2 (Severity)	COVID-19 B2 (Severity)	COVID-19 C2 (Susceptibility)
Univariable MR analysis (after Bonferroni correction)	Causative (risk *)	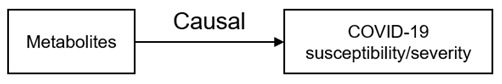	Credible	2-stearoylglycerophosphocholine (↑)	bradykinin-des-arg(9) (↑) decanoylcarnitine (↑) thymol sulfate (↑)	—
			Suggestive	—	1-heptadecanoylglycerophosphocholine (↑) glutamate (↑)	—
Mediation analysis	Mediator of T2D (direction ^†^)	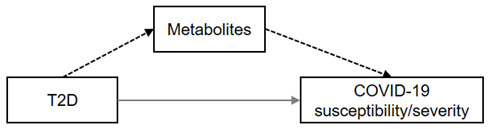	Strong	—	gamma-glutamyltyrosine (+)	—
			Moderate	—	glutamate (+)	glutamate (−)
			Potential	valine (+) indoleacetate (−) lactate (−) fructose (−) heptanoate (7:0) (+)	Indoleacetate (−) Inosine (−)	gamma-glutamyltyrosine (+)
	Mediator of BMI (direction ^†^)	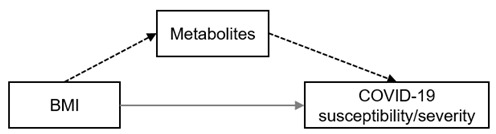	Strong	valine (+)	gamma-glutamyltyrosine (+)	—
			Moderate	heptanoate (7:0) (+) alpha-glutamyltyrosine (−)	glutamate (+)	glutamate (−) alpha-glutamyltyrosine (−) gamma-glutamyltyrosine (+)
			Potential	1-oleoylglycerophosphocholine (+) taurochenodeoxycholate (−) 2-stearoylglycerophosphocholine (−) 2−tetradecenoyl carnitine (−)	bradykinin-des-arg(9) (+) xanthine (−)	phenol sulfate (−) propionylcarnitine (−) 2−tetradecenoyl carnitine (−) quinate (−)

*: indicator of effect direction, where “↑” suggests that higher serum levels of the metabolite would increase the risk of the corresponding outcome. †: indicator of the direction of mediation effect, where “+” suggests that the mediation effect is in the same direction as the total effect of T2D/BMI (i.e., risk-conferring), while “−” suggests that the mediation effect is in the opposite direction from the total effect (i.e., risk-reducing).

**Table 2:** Mediation effects of human serum metabolites in the total effect between T2D and COVID-19 phenotypes.

Mediator	Category	Exposure	Outcome	Beta*_XM_*	OR*_MY_*	OR*_XY_*	Beta*_indirect_* (95% CI)	Proportion Mediated (95% CI)	MVMR
valine	Amino acid	T2D	A2	0.006	3.516	1.057	0.008 (−0.001, 0.016)	— *	F ^†^
indoleacetate	Amino acid	T2D	A2	0.016	0.670		−0.006 (−0.014, 0.002)	—	F
lactate	Carbohydrate	T2D	A2	0.011	0.483		−0.008 (−0.018, 0.002)	—	F
fructose	Carbohydrate	T2D	A2	0.023	0.505		−0.016 (−0.03, −0.001)	—	F
heptanoate (7:0)	Lipid	T2D	A2	−0.014	0.454		0.011 (−0.001, 0.022)	—	F
indoleacetate	Amino acid	T2D	B2	0.016	0.784	1.054	−0.004 (−0.008, 0.001)	—	F
glutamate	Amino acid	T2D	B2	0.027	1.393		**0.009 (0.002, 0.015)**	**16.78% (1.04%, 32.52%)**	F
inosine	Nucleotide	T2D	B2	−0.054	1.094		−0.005 (−0.01, 0.001)	—	F
gamma-glutamyltyrosine	Peptide	T2D	B2	0.016	1.745		**0.009 (0.002, 0.016)**	**16.67% (0.64%, 32.70%)**	**P**
glutamate	Amino acid	T2D	C2	0.027	0.877	1.019	−0.004 (−0.006, −0.001)	—	**P**
gamma-glutamyltyrosine	Peptide	T2D	C2	0.016	1.228		0.003 (0.001, 0.006)	17.63% (−2.83%, 38.08%)	F

T2D: type 2 diabetes; Beta*_XM_*: causal effect estimates of exposure on the mediator; OR*_MY_*: causal effect estimates of the mediator on the outcome in odds ratio scale; OR*_XY_*: total effect estimates of exposure on the outcome in odds ratio scale; Beta*_indirect_*: indirect effect estimates of exposure on the outcome mediated by the mediator in log OR scale; CI: confidence interval. MVMR: multivariable Mendelian randomization analysis. *: Proportion of mediation effects was not calculated for indirect effects (Beta*_indirect_*) in the opposite direction from the total effect (OR*_XY_*) or without statistical significance. Mediation effects supported by both significant Beta*_indirect_* and proportion mediated are in boldface type. †: Indicator of statistical significance of MVMR analysis for casual associations between mediators and COVID-19 phenotypes conditioning on genetically predicted T2D risk. “P” in boldface type suggests that the mediator–outcome association remained significant in the MVMR analysis, while “F” suggests that the association failed to keep significance after T2D adjustment.

**Table 3:** Mediation effects of human serum metabolites in the total effect between obesity and COVID-19 phenotypes.

Mediator	Category	Exposure	Outcome	Beta*_XM_*	OR*_MY_*	OR*_XY_*	Beta*_indirect_* (95% CI)	Proportion Mediated (95% CI)	MVMR
valine	Amino acid	BMI	A2	0.014	3.516	1.698	**0.018 (0.001, 0.036)**	**4.19% (0.03%, 8.35%)**	**P ^†^**
heptanoate (7:0)	Lipid	BMI	A2	−0.031	0.454		**0.025 (0.001, 0.049)**	**5.75% (0.20%, 11.30%)**	F
taurochenodeoxycholate	Lipid	BMI	A2	0.076	0.78		−0.019 (−0.043, 0.005)	— *	F
1-oleoylglycerophosphocholine	Lipid	BMI	A2	−0.031	0.486		0.022 (−0.003, 0.048)	—	F
2-stearoylglycerophosphocholine	Lipid	BMI	A2	−0.038	2.145		−0.029 (−0.058, 0.001)	—	F
2-tetradecenoyl carnitine	Lipid	BMI	A2	−0.046	1.441		−0.017 (−0.038, 0.004)	—	F
alpha-glutamyltyrosine	Peptide	BMI	A2	−0.063	1.322		−0.018 (−0.039, 0.004)	—	**P**
glutamate	Amino acid	BMI	B2	0.051	1.393	1.523	**0.017 (0.002, 0.031)**	**4.59% (0.69%, 8.49%)**	F
xanthine	Nucleotide	BMI	B2	0.039	0.671		−0.016 (−0.033, 0.001)	—	F
gamma-glutamyltyrosine	Peptide	BMI	B2	0.041	1.745		**0.023 (0.006, 0.04)**	**6.32% (1.76%, 10.87%)**	**P**
bradykinin, des-arg(9)	Peptide	BMI	B2	0.109	1.087		0.009 (−0.001, 0.019)	—	F
glutamate	Amino acid	BMI	C2	0.051	0.877	1.141	**−0.007 (−0.013, −0.00016)**	—	**P**
phenol sulfate	Amino acid	BMI	C2	−0.06	1.08		−0.005 (−0.01, 0.001)	—	F
propionylcarnitine	Lipid	BMI	C2	0.026	0.896		−0.003 (−0.006, 0.001)	—	F
2-tetradecenoyl carnitine	Lipid	BMI	C2	−0.046	1.058		−0.003 (−0.006, 0.001)	—	F
gamma-glutamyltyrosine	Peptide	BMI	C2	0.041	1.228		**0.009 (0.002, 0.015)**	**6.62% (1.52%, 11.71%)**	F
alpha-glutamyltyrosine	Peptide	BMI	C2	−0.063	1.093		−0.006 (−0.012, 0.001)	—	**P**
quinate	Xenobiotics	BMI	C2	0.123	0.956		−0.006 (−0.012, 0.001)	—	F

BMI: body mass index; Beta*_XM_*: causal effect estimates of exposure on the mediator; OR*_MY_*: causal effect estimates of the mediator on the outcome in odds ratio scale; OR*_XY_*: total effect estimates of exposure on the outcome in odds ratio scale; Beta*_indirect_*: indirect effect estimates of exposure on the outcome mediated by the mediator in log OR scale; CI: confidence interval. MVMR: multivariable Mendelian randomization analysis. *: Proportion of mediation effects was not calculated for indirect effects (Beta*_indirect_*) in the opposite direction from the total effect (OR*_XY_*) or without statistical significance. Mediation effects supported by both significant Beta*_indirect_* and proportion mediated are in boldface type. †: Indicator of statistical significance of MVMR analysis for casual associations between mediators and COVID-19 phenotypes conditioning on genetically predicted BMI. “P” in boldface type suggests that the mediator–outcome association remained significant in the MVMR analysis, while “F” suggests that the association failed to keep significance after BMI adjustment.

## Data Availability

Restrictions apply to the availability of these data. Data was obtained from GWAS summary statistics for type 2 diabetes are available at: https://www.diagram-consortium.org/ (accessed on 30 December 2021). GWAS summary statistics for obesity are available at: https://portals.broadinstitute.org/collaboration/giant/index.php/GIANT_consortium (accessed on 30 December 2021). GWAS summary statistics for glycemic traits are available at: https://magicinvestigators.org/ (accessed on 30 December 2021). GWAS summary statistics for human serum metabolites are available at: http://metabolomics.helmholtz-muenchen.de/gwas/ (accessed on 30 December 2021). GWAS summary statistics for COVID-19 phenotypes are available at: https://www.covid19hg.org/ (accessed on 30 December 2021). The datasets analyzed in this study are publicly available summary statistics.

## Supplementary Materials

The following supporting information can be downloaded at: https://www.mdpi.com/article/10.3390/metabo12070598/s1, Supplementary Material Figure S1. Causal associations between metabolic traits and COVID-19 phenotypes, Supplementary Material Figure S2. Relationship of the SNP (instrument variable) effects on the metabolite serum levels with the SNP effects on the COVID-19 phenotypes estimated by three different MR methods for the six metabolites that passed the Bonferroni correction (PIVW < 0.05/302 = 1.66 × 10−4), Supplementary Material Figure S3. Residual associations between T2D-associated human serum metabolites and COVID-19 phenotypes after adjusting for T2D, Supplementary Material Figure S4. Residual associations between BMI-associated human serum metabolites and COVID-19 phenotypes after adjusting for BMI, Supplementary Material Table S1. GWAS summary data used in the current study, Supplementary Material Table S2. Instrument strength for the 56 metabolites causally associated with COVID-19 outcomes at nominal significance (PIVW < 0.05), Supplementary Material Table S3, (a) Association between genetically predicted metabolic traits and COVID-19 phenotypes, (b) Assessment of the horizontal pleiotropic effects between metabolic traits and COVID-19 phenotypes, Supplementary Material Table S4. Causal associations between human serum metabolites and COVID-19 phenotypes from metabolite–outcome screening, Supplementary Material Table S5. Assessment of the causal relationship between T2D and metabolites (T2D as exposure), Supplementary Material Table S6. Direct effects of T2D-associated metabolites on COVID-19 phenotypes conditioning on T2D, Supplementary Material Table S7. Assessment of the causal relationship between BMI and metabolites (BMI as exposure), Supplementary Material Table S8. Direct effects of BMI-associated metabolites on COVID-19 phenotypes conditioning on BMI, Supplementary Material Table S9. Association between metabolites and COVID-19 phenotypes after adjusting for other metabolites with correlated serum levels.
