# Supplementary material for: Human Serum Metabolites as Potential Mediators from Type 2 Diabetes and Obesity to COVID-19 Severity and Susceptibility: Evidence from Mendelian Randomization Study
Source: Metabolites. 2022 Jun 27;12(7):598. doi: 10.3390/metabo12070598 (PMC9319376; doi:10.3390/metabo12070598)
Supplement: Supplementary file 1 [file metabolites-12-00598-s001.zip › metabolites-1771056-supplementary.pdf]

Human Serum Metabolites as Potential Mediators from Type 2 Diabetes and Obesity to COVID-19 Severity and Susceptibility: Evidence from Mendelian Randomization Study

Huang et al.

Supplementary Figures and Tables

Contents

Supplementary Figure S1. 2
Supplementary Figure S2. 4
Supplementary Figure S3. 5
Supplementary Figure S4. 6
Supplementary Table S1. 7
Supplementary Table S2. 8
Supplementary Table S3. 10
Supplementary Table S4. 12
Supplementary Table S5. 15
Supplementary Table S6. 17
Supplementary Table S7. 18
Supplementary TableS 8. 20
Supplementary Table S9. 21

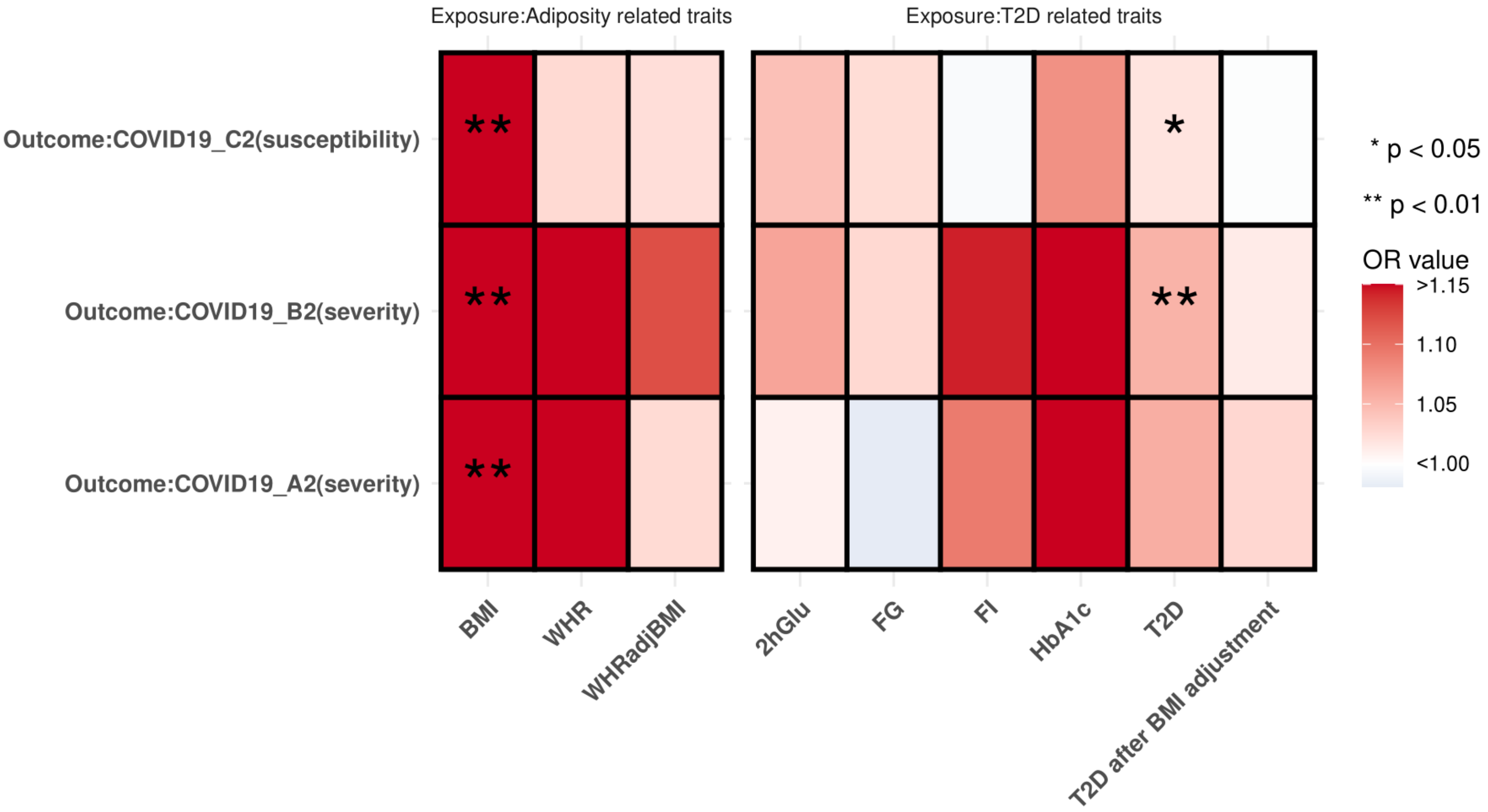

Supplementary Figure S1. Causal associations between metabolic traits and COVID-19 phenotypes. BMI: body mass index; WHR: waist-hip-ratio; WHRadjBMI: waist-hip-ratio with BMI adjustment; 2hGlu: 2h-glucose after an oral glucose challenge; FG: fasting glucose; HbA1c: glycated hemoglobin; T2D: type 2 diabetes; OR: odds ratio. The reported effect estimates and statistical significance were derived from the inverse-variance weighted method with multiplicative random-effects model. Number of asterisks indicates the level of statistical significance: ** $P_{\mathrm{IVW}} < 0.01$; * $P_{\mathrm{IVW}} < 0.05$.

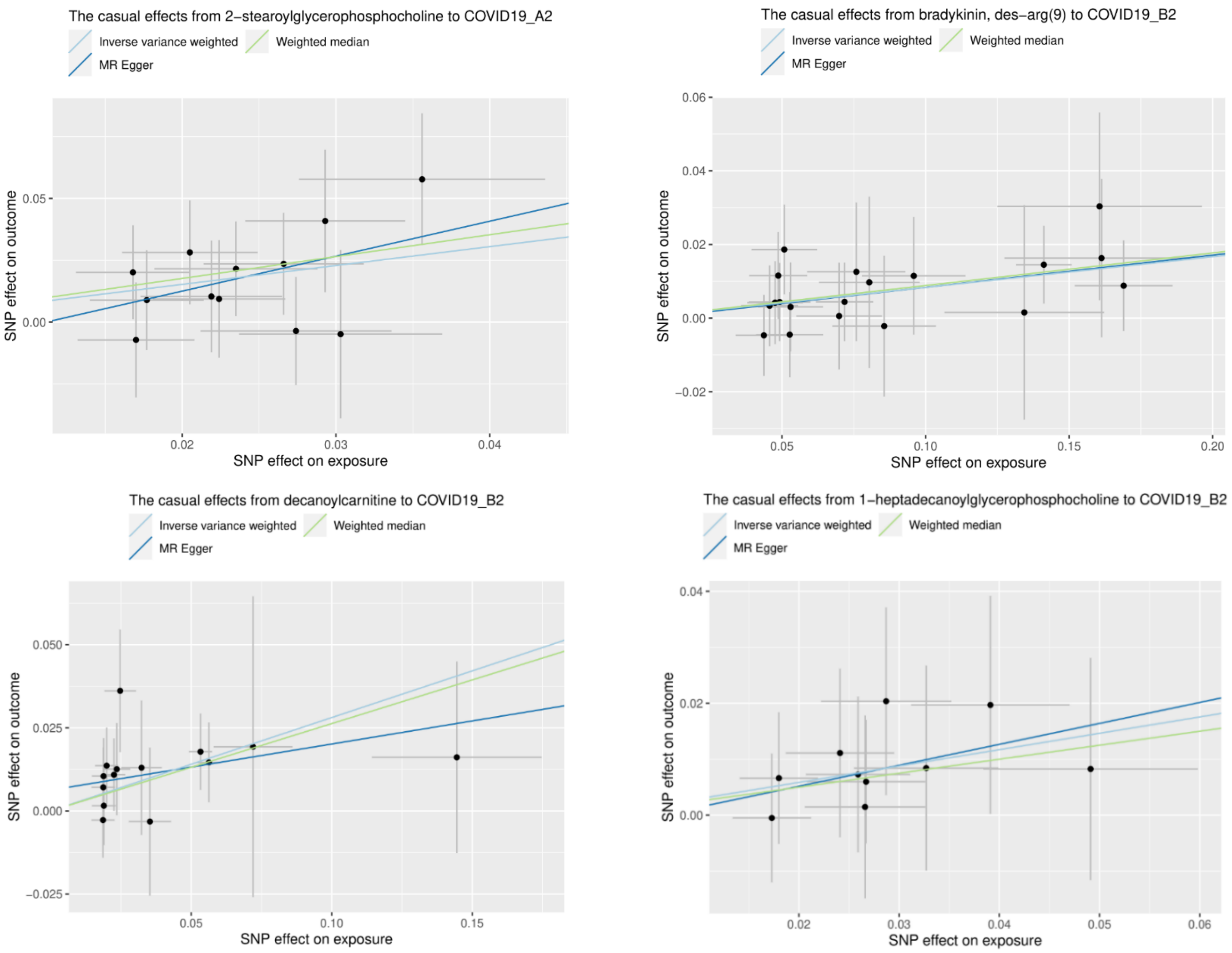
The casual effects from 2−stearoylglycerophosphocholine to COVID19_A2
Inverse variance weighted
Weighted median
MR Egger
SNP effect on outcome
SNP effect on exposure
The casual effects from bradykinin, des−arg(9) to COVID19_B2
Inverse variance weighted
Weighted median
MR Egger
SNP effect on outcome
SNP effect on exposure
The casual effects from decanoylcarnitine to COVID19_B2
Inverse variance weighted
Weighted median
MR Egger
SNP effect on outcome
SNP effect on exposure
The casual effects from 1−heptadecanoylglycerophosphocholine to COVID19_B2
Inverse variance weighted
Weighted median
MR Egger
SNP effect on outcome
SNP effect on exposure

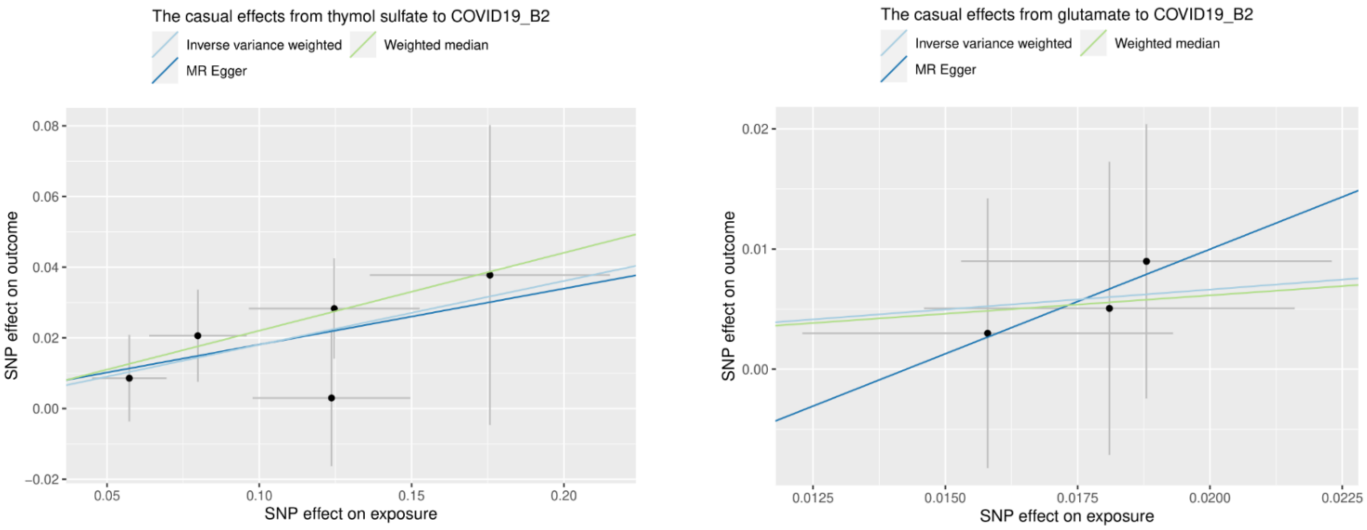

Supplementary Figure S2. Relationship of the SNP (instrument variable) effects on the metabolite serum levels against the SNP effects on the COVID-19 phenotypes estimated by three different MR methods for the six metabolites that passed the Bonferroni correction ($P_{\mathrm{IVW}} < 0.05/302 = 1.66\times10^{-4}$).

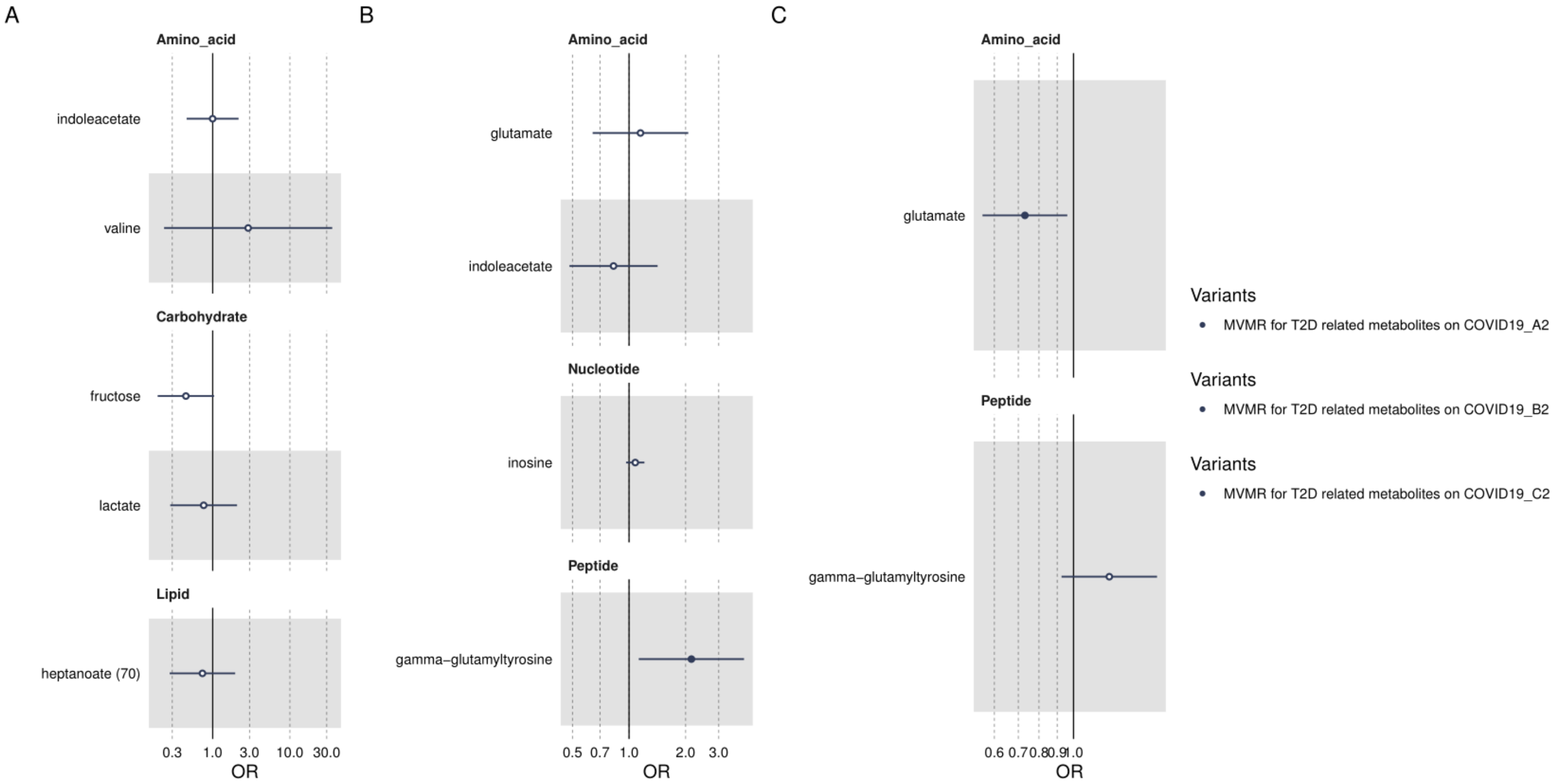

Supplementary Figure S3. Residual associations between T2D-associated human serum metabolites and COVID-19 phenotypes after adjusting for T2D. X-axis was the direct odds ratio of outcome per 1-log10 unit increase of the serum levels of metabolites conditioning on T2D. Solid dots indicate significant associations ($P_{MVMR} < 0.05$) (**A**) Multivariable MR (MVMR) for T2D related metabolites on COVID-19 A2 phenotype, (**B**) Multivariable MR (MVMR) for T2D related metabolites on COVID-19 B2 phenotype, (**C**) Multivariable MR (MVMR) for T2D related metabolites on COVID-19 C2 phenotype.

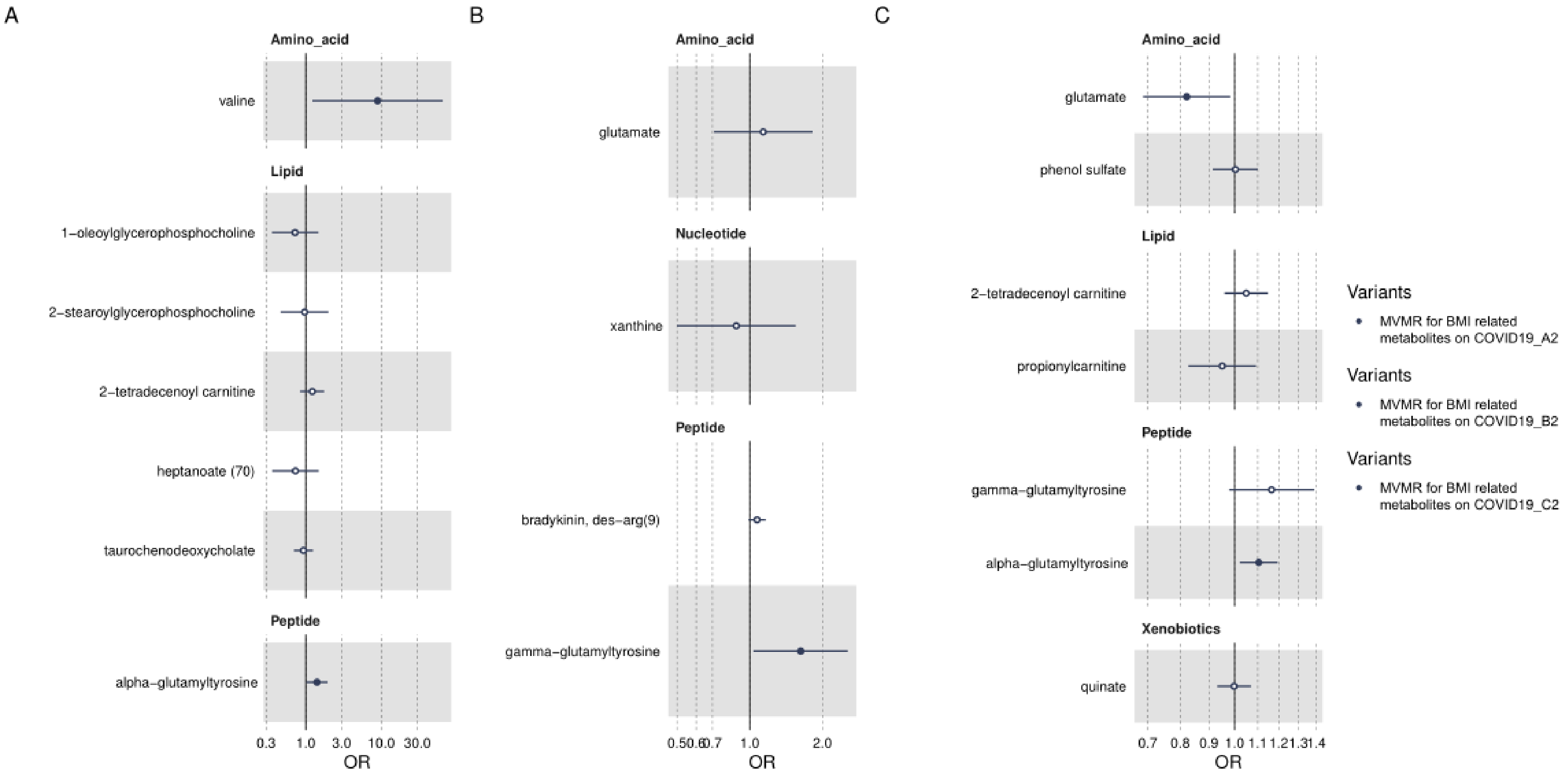

Supplementary Figure S4. Residual associations between BMI-associated human serum metabolites and COVID-19 phenotypes after adjusting for BMI. X-axis was the direct odds ratio of outcome per 1-log10 unit increase of the serum levels of metabolites conditioning on BMI. Solid dots indicate significant associations ($P_{MVMR} < 0.05$) (**A**) Multivariable MR (MVMR) for BMI related metabolites on COVID-19 A2 phenotype, (**B**) Multivariable MR (MVMR) for BMI related metabolites on COVID-19 B2 phenotype, (**C**) Multivariable MR (MVMR) for BMI related metabolites on COVID-19C2 phenotype.

Supplementary Table S1. GWAS summary data used in the current study.

Traits	Traits Category	Number of Participants Included in Original GWAS	Population	Data Source
Type 2 diabetes	Exposure/Mediator	74,124 (cases) vs. 824,006 (controls)	European	DIAGRAM consortium [1]
Glycemic traits	Exposure/Mediator	Individuals without diabetes;<br>fasting glucose (FG): 200,622<br>2h-glucose post-challenge (2hGlu): 63,396<br>glycated haemoglobin (HbA1c): 146,806<br>fasting insulin (FI): 151,013	European	MAGIC consortium [2]
Adiposity traits	Exposure/Mediator	Body mass index (BMI): 322,154<br>Waist-hip-ratio (WHR): 212,248<br>Waist-hip-ratio adjusted for BMI (WHRadjBMI): 210,086	European	GIANT consortium GWAS (Anthropometric 2015) [3]
Human serum metabolites	Exposure/Mediator	7,824 healthy adult participants	European	KORA + TwinsUK [4]
COVID19 (A2)	Outcome	Very severe respiratory confirmed COVID-19 (n = 8,779) vs. general population (n = 1,001,875)	Mixed	COVID-19 Host Genetics Initiative [5]
COVID19 (B2)	Outcome	Hospitalized COVID-19 (n = 24,274) vs. general population (n = 2,061,529)	Mixed	
COVID19 (C2)	Outcome	Laboratory-confirmed SARS-CoV-2 infection OR coding/physician-confirmed COVID-19 OR self-reported COVID-19 via questionnaire (n = 112,612) vs. general population (n = 2,474,079)	Mixed	

[1] http://diagram-consortium.org/

[2] https://magicinvestigators.org/

[3] https://portals.broadinstitute.org/collaboration/giant/index.php/GIANT_consortium_data_files

[4] http://mips.helmholtz-muenchen.de/proj/GWAS/gwas/index.php

[5] https://www.covid19hg.org/, round 6, released on June 15, 2021

Supplementary Table S2. Instrument strength for the 56 metabolites causally associated with COVID-19 outcomes at nominal significance (P_{IVW} <0.05).

Metabolite_ID	Category	Metabolite	Outcomes								
			COVID-19_A2			COVID-19_B2			COVID-19_C2		
			Nsnp	R^2	F	Nsnp	R^2	F	Nsnp	R^2	F
M00059	Amino_acid	histidine	6	0.044	56.603	7	0.055	61.377	7	0.055	61.377
M01284	Amino_acid	threonine	19	0.080	25.935	19	0.080	25.935	19	0.080	25.935
M01585	Amino_acid	N-acetylalanine	22	0.066	23.466	23	0.069	23.500	23	0.069	23.500
M01649	Amino_acid	valine	4	0.015	28.829	6	0.041	52.554	6	0.041	52.554
M22030	Amino_acid	2-hydroxyisobutyrate	19	0.082	27.603	19	0.082	27.603	19	0.082	27.603
M27513	Amino_acid	indoleacetate	20	0.085	31.625	20	0.085	31.625	20	0.085	31.625
M32322	Amino_acid	glutamate	3	0.013	24.243	3	0.013	24.243	3	0.013	24.243
M32553	Amino_acid	phenol sulfate	13	0.056	33.258	13	0.056	33.258	13	0.056	33.258
M33441	Amino_acid	isobutyrylcarnitine	10	0.074	58.383	10	0.074	58.383	10	0.074	58.383
M33515	Amino_acid	hydroxytryptophan	42	0.147	28.197	42	0.147	28.197	42	0.147	28.197
M00527	Carbohydrate	lactate	12	0.054	35.144	13	0.057	34.414	13	0.057	34.414
M00577	Carbohydrate	fructose	3	0.009	22.524	3	0.009	22.524	3	0.009	22.524
M27738	CVs*	threonate	18	0.097	43.478	18	0.097	43.478	18	0.097	43.478
M01303	Energy	malate	17	0.066	28.948	17	0.066	28.948	17	0.066	28.948
M11438	Energy	phosphate	4	0.039	75.079	4	0.039	75.079	5	0.042	64.604
M33453	Energy	alpha-ketoglutarate	18	0.096	31.729	18	0.096	31.729	18	0.096	31.729
M00542	Lipid	3-hydroxybutyrate (BHBA)	10	0.058	45.028	10	0.058	45.028	10	0.058	45.028
M01105	Lipid	linoleate (18:2n6)	17	0.051	23.236	17	0.051	23.236	17	0.051	23.236
M01644	Lipid	heptanoate (7:0)	33	0.123	30.314	33	0.123	30.314	34	0.126	30.241
M15500	Lipid	carnitine	160	0.620	70.733	160	0.623	71.137	160	0.623	71.137
M15990	Lipid	glycerophosphorylcholine (GPC)	18	0.091	37.768	18	0.091	37.768	18	0.091	37.768
M18494	Lipid	taurochenodeoxycholate	12	0.080	39.005	12	0.080	39.005	13	0.099	45.286
M22189	Lipid	palmitoylcarnitine	8	0.037	31.014	10	0.040	30.135	10	0.040	30.135
M27531	Lipid	hyodeoxycholate	15	0.121	48.733	16	0.125	47.683	16	0.125	47.683
M32388	Lipid	dodecanedioate	6	0.083	90.905	7	0.086	81.253	7	0.086	81.253
M32452	Lipid	propionylcarnitine	30	0.160	43.615	30	0.163	43.159	30	0.163	43.159
M33230	Lipid	1-palmitoleoylglycerophosphocholine	14	0.045	23.118	14	0.045	23.118	14	0.045	23.118
M33447	Lipid	palmitoleate (16:1n7)	9	0.053	37.510	9	0.053	37.510	9	0.053	37.510
M33821	Lipid	1-eicosatrienoylglycerophosphocholine	19	0.091	38.545	19	0.091	38.545	20	0.094	37.911

M33941	Lipid	decanoylcarnitine	14	0.094	54.389	14	0.094	54.389	14	0.094	54.389
M33957	Lipid	1-heptadecanoylglycerophosphocholine	10	0.039	28.291	10	0.039	28.291	10	0.039	28.291
M33960	Lipid	1-oleoylglycerophosphocholine	14	0.050	27.838	16	0.056	27.197	16	0.056	27.197
M33973	Lipid	epiandrosterone sulfate	12	0.094	63.518	12	0.094	63.518	12	0.094	63.518
M35160	Lipid	oleoylcarnitine	11	0.046	29.432	12	0.046	29.432	12	0.046	29.432
M35186	Lipid	1-arachidonoylglycerophosphoethanolamine	21	0.105	38.950	21	0.105	38.950	21	0.105	38.950
M35189	Lipid	nonanoylcarnitine	13	0.139	77.361	13	0.139	77.361	13	0.139	77.361
M35255	Lipid	2-stearoylglycerophosphocholine	12	0.073	47.967	13	0.077	46.780	13	0.077	46.780
M35472	Lipid	2-tetradecenoyl carnitine	19	0.110	42.901	19	0.110	42.901	19	0.110	42.901
M35626	Lipid	1-myristoylglycerophosphocholine	6	0.034	43.633	6	0.034	43.633	6	0.034	43.633
M36754	Lipid	octadecanedioate	9	0.060	48.755	9	0.060	48.755	9	0.060	48.755
M36850	Lipid	taurolithocholate 3-sulfate	10	0.063	43.693	10	0.063	43.693	10	0.063	43.693
M37203	Lipid	4-androsten-3beta,17beta-diol disulfate 2	18	0.066	28.933	18	0.066	28.933	18	0.066	28.933
M01123	Nucleotide	inosine	10	0.113	33.599	10	0.113	33.599	10	0.113	33.599
M03127	Nucleotide	hypoxanthine	21	0.096	33.246	22	0.096	33.246	22	0.096	33.246
M32739	Nucleotide	xanthine	7	0.067	47.883	7	0.067	47.883	7	0.067	47.883
M02734	Peptide	gamma-glutamyltyrosine	44	0.171	31.323	44	0.171	31.323	44	0.171	31.323
M18357	Peptide	glycylvaline	7	0.078	24.929	7	0.078	24.929	7	0.078	24.929
M34420	Peptide	bradykinin, des-arg(9)	18	0.172	51.190	19	0.177	50.100	19	0.177	50.100
M36131	Peptide	alpha-glutamyltyrosine	15	0.216	32.411	15	0.216	32.411	15	0.216	32.411
M36738	Peptide	gamma-glutamylglutamate	10	0.301	39.225	10	0.301	39.225	10	0.301	39.225
M36756	Peptide	leucylleucine	11	0.087	28.135	11	0.087	28.135	11	0.087	28.135
M01515	Xenobiotics	salicylate	18	0.399	83.746	18	0.399	83.746	18	0.399	83.746
M18335	Xenobiotics	quinate	6	0.036	36.237	6	0.036	36.237	6	0.036	36.237
M18392	Xenobiotics	theobromine	6	0.018	22.168	6	0.018	22.168	6	0.018	22.168
M21151	Xenobiotics	saccharin	10	0.168	46.870	10	0.168	46.870	10	0.168	46.870
M36095	Xenobiotics	thymol sulfate	5	0.054	45.244	5	0.054	45.244	5	0.054	45.244

*: cofactors and vitamins. Nsnp: number of valid genetic instruments after Steiger filtering.

The full list of the instrument strength for all the 302 metabolites is available at https://doi.org/10.6084/m9.figshare.18737738.

Supplementary Table S3. (a) Association between genetically predicted metabolic traits and COVID-19 phenotypes. (b) Assessment of the horizontal pleiotropic effects between metabolic traits and COVID-19 phenotypes.

(a)

Exposure	MR Method	COVID-19 A2 (Severity)		COVID-19 B2 (Severity)		COVID-19 C2 (Susceptibility)	
		**OR (95% CI)**	***P***	**OR (95% CI)**	***P***	**OR (95% CI)**	***P***
T2D							
	Inverse variance weighted*	1.06 (1.00, 1.12)	0.055	**1.05 (1.01, 1.10)**	**0.007**	**1.02 (1.00, 1.04)**	**0.039**
	Weighted median	1.00 (0.92, 1.10)	0.960	1.05 (0.99, 1.10)	0.094	1.02 (1.00, 1.04)	0.093
	MR Egger	0.90 (0.79, 1.03)	0.117	0.94 (0.87, 1.03)	0.190	0.98 (0.94, 1.02)	0.296
	MR-PRESSO (outlier-corrected)	1.05 (0.99, 1.10)	0.088	**1.05 (1.02, 1.09)**	**0.003**	**1.01 (1.00, 1.02)**	**0.030**
T2DadjBMI							
	Inverse variance weighted	1.03 (0.97, 1.08)	0.314	1.01 (0.98, 1.05)	0.408	1.00 (0.99, 1.01)	0.806
	Weighted median	0.97 (0.89, 1.06)	0.468	1.04 (0.99, 1.09)	0.148	1.01 (0.99, 1.03)	0.376
	MR Egger	0.93 (0.82, 1.05)	0.247	0.99 (0.92, 1.07)	0.755	1.00 (0.97, 1.02)	0.768
	MR-PRESSO (outlier-corrected)	-†	-	1.01 (0.98, 1.04)	0.512	-	-
FG							
	Inverse variance weighted	0.98 (0.70, 1.37)	0.911	1.03 (0.84, 1.26)	0.799	1.02 (0.92, 1.14)	0.660
	Weighted median	0.91 (0.64, 1.30)	0.598	1.01 (0.81, 1.25)	0.939	1.02 (0.93, 1.11)	0.685
	MR Egger	0.71 (0.38, 1.34)	0.298	0.90 (0.61, 1.34)	0.616	1.00 (0.81, 1.23)	0.997
	MR-PRESSO (outlier-corrected)	0.88 (0.68, 1.14)	0.332	0.96 (0.82, 1.11)	0.569	0.99 (0.94, 1.05)	0.808
FI							
	Inverse variance weighted	1.09 (0.74, 1.62)	0.662	1.14 (0.89, 1.48)	0.304	1.00 (0.89, 1.12)	0.951
	Weighted median	1.47 (0.77, 2.79)	0.243	1.18 (0.81, 1.73)	0.384	0.98 (0.85, 1.14)	0.830
	MR Egger	0.87 (0.23, 3.33)	0.842	0.51 (0.24, 1.09)	0.089	0.78 (0.55, 1.10)	0.163
	MR-PRESSO (outlier-corrected)	-	-	-	-	0.98 (0.88, 1.09)	0.667
HbA1c							
	Inverse variance weighted	1.37 (0.86, 2.20)	0.187	1.26 (0.95, 1.67)	0.108	1.08 (0.94, 1.23)	0.277
	Weighted median	1.29 (0.75, 2.24)	0.360	1.21 (0.88, 1.67)	0.242	1.01 (0.91, 1.14)	0.806
	MR Egger	1.03 (0.43, 2.48)	0.943	1.27 (0.76, 2.13)	0.372	0.99 (0.77, 1.27)	0.944
	MR-PRESSO (outlier-corrected)	1.35 (0.92, 1.99)	0.132	**1.26 (1.04, 1.54)**	**0.022**	1.04 (0.96, 1.12)	0.336
2hGlu							
	Inverse variance weighted	1.01 (0.70, 1.44)	0.960	1.06 (0.83, 1.36)	0.622	1.04 (0.87, 1.25)	0.634
	Weighted median	0.87 (0.69, 1.10)	0.255	0.98 (0.85, 1.12)	0.773	0.96 (0.91, 1.01)	0.112
	MR Egger	0.51 (0.20, 1.28)	0.182	0.82 (0.41, 1.65)	0.588	0.79 (0.49, 1.28)	0.362
	MR-PRESSO (outlier-corrected)	0.90 (0.77, 1.05)	0.215	0.97 (0.91, 1.04)	0.417	0.98 (0.94, 1.01)	0.170
BMI							
	Inverse variance weighted	**1.70 (1.39, 2.07)**	**<0.001**	**1.52 (1.33, 1.75)**	**<0.001**	**1.14 (1.08, 1.20)**	**<0.001**
	Weighted median	**1.65 (1.24, 2.18)**	**<0.001**	**1.58 (1.34, 1.87)**	**<0.001**	**1.14 (1.08, 1.22)**	**<0.001**
	MR Egger	1.59 (0.88, 2.87)	0.127	**1.61 (1.08, 2.41)**	**0.023**	**1.22 (1.04, 1.43)**	**0.019**
	MR-PRESSO (outlier-corrected)	**-**	**-**	**-**	**-**	**1.13 (1.07, 1.18)**	**<0.001**
WHR							
	Inverse variance weighted	1.22 (0.85, 1.77)	0.283	1.22 (0.99, 1.50)	0.056	1.03 (0.96, 1.09)	0.417
	Weighted median	1.22 (0.78, 1.92)	0.382	1.11 (0.85, 1.45)	0.438	1.06 (0.97, 1.17)	0.206

	MR Egger	1.18 (0.22, 6.40)	0.850	0.78 (0.31, 1.98)	0.606	1.03 (0.76, 1.40)	0.841
	MR-PRESSO (outlier-corrected)	-	-	-	-	-	-
WHRadjBMI							
	Inverse variance weighted	1.03 (0.76, 1.38)	0.867	1.12 (0.96, 1.31)	0.144	1.02 (0.96, 1.09)	0.441
	Weighted median	0.91 (0.64, 1.29)	0.591	1.14 (0.92, 1.40)	0.230	1.05 (0.97, 1.13)	0.203
	MR Egger	1.12 (0.28, 4.51)	0.870	0.81 (0.39, 1.66)	0.565	1.02 (0.76, 1.35)	0.917
	MR-PRESSO (outlier-corrected)	1.02 (0.81, 1.29)	0.868	-	-	-	-

(b)

Exposure	Outcome	MR–PRESSO Global Test		MR–Egger Regression		
		RSSobs*	***P***	**Intercept**	**SE**	***P***
T2D	COVID-19_A2	**246.200**	**0.003**	**0.011**	**0.004**	**0.009**
T2D	COVID-19_B2	**366.210**	**<0.001**	**0.008**	**0.003**	**0.006**
T2D	COVID-19_C2	**530.796**	**<0.001**	**0.003**	**0.001**	**0.029**
T2DadjBMI	COVID-19_A2	171.183	0.059	0.009	0.005	0.081
T2DadjBMI	COVID-19_B2	213.829	0.168	0.002	0.003	0.456
T2DadjBMI	COVID-19_C2	139.884	0.556	0.000	0.001	0.835
FG	COVID-19_A2	130.984	0.611	0.009	0.007	0.248
FG	COVID-19_B2	160.440	0.224	0.003	0.005	0.455
FG	COVID-19_C2	286.596	0.111	0.001	0.002	0.783
FI	COVID-19_A2	32.702	0.764	0.004	0.011	0.730
FI	COVID-19_B2	42.055	0.366	**0.014**	**0.006**	**0.034**
FI	COVID-19_C2	**56.952**	**0.031**	0.004	0.003	0.146
HbA1c	COVID-19_A2	**142.832**	**<0.001**	0.006	0.007	0.450
HbA1c	COVID-19_B2	**166.754**	**<0.001**	0.000	0.004	0.971
HbA1c	COVID-19_C2	**258.324**	**<0.001**	0.002	0.002	0.434
2hGlu	COVID-19_A2	**52.097**	**<0.001**	0.053	0.034	0.150
2hGlu	COVID-19_B2	**83.566**	**<0.001**	0.020	0.025	0.450
2hGlu	COVID-19_C2	**304.964**	**<0.001**	0.021	0.018	0.251
BMI	COVID-19_A2	77.259	0.237	0.002	0.008	0.818
BMI	COVID-19_B2	110.062	0.067	-0.002	0.006	0.767
BMI	COVID-19_C2	115.240	0.525	-0.002	0.002	0.407
WHR	COVID-19_A2	42.835	0.072	0.001	0.022	0.966
WHR	COVID-19_B2	41.315	0.082	0.012	0.012	0.342
WHR	COVID-19_C2	25.158	0.679	0.000	0.004	0.971
WHRadjBMI	COVID-19_A2	55.319	0.959	-0.003	0.020	0.895
WHRadjBMI	COVID-19_B2	45.307	0.161	0.010	0.011	0.368
WHRadjBMI	COVID-19_C2	44.507	0.195	0.000	0.004	0.956

In part (a), T2D: type 2 diabetes; BMI: body mass index; T2DadjBMI: T2D with BMI adjustment; FG: fasting glucose; FI: fasting insulin; HbA1c: glycated hemoglobin; 2hGlu: 2-hour plasma glucose after a 75-gram oral glucose tolerance test; WHR: waist-hip ratio; WHRadjBMI: WHR with BMI adjustment; MR-PRESSO: MR-Pleiotropy Residual Sum and Outlier; OR: odds ratio; CI: confidence interval. Associations of nominal significance are in **boldface** type. *: Inverse variance weighted method with multiplicative random-effects

model was used as the primary method in the two-sample MR analysis. †: No outlier was detected. In part (b), *: the *RSSobs* in MR–PRESSO global test is equivalent to the Cochran's Q statistic. Significant results are in **boldface** type, which suggest the **existence** of heterogeneity ($P_{\text{MR-PRESSO Global}}$ <0.05) or horizontal pleiotropy ($P_{\text{MR-Egger intercept}}$ <0.05).

Supplementary Table S4. Causal associations between human serum metabolites and COVID-19 phenotypes from metabolite-outcome screening.

COVID-19 Outcome	Category	Metabolite	IVW Method		Weighted Median Method		MR-Egger Regression	
			OR (95% CI)	*P*	OR (95% CI)	*P*	Intercept	*P*
A2 (severity)								
	Amino_acid	histidine	3.99 (1.11, 14.36)	0.034	4.74 (0.35, 64.29)	0.242	-0.007	0.739
	Amino_acid	valine	3.52 (1.69, 7.33)	0.001	2.87 (0.08, 100.01)	0.561	0.022	0.756
	Amino_acid	indoleacetate	0.67 (0.48, 0.94)	0.019	0.73 (0.38, 1.37)	0.323	0.004	0.651
	Amino_acid	hydroxytryptophan	0.57 (0.34, 0.95)	0.033	0.63 (0.25, 1.57)	0.321	0.012	0.374
	Carbohydrate	lactate	0.48 (0.24, 0.95)	0.035	0.78 (0.22, 2.73)	0.692	0.013	0.547
	Carbohydrate	fructose	0.50 (0.33, 0.76)	0.001	0.44 (0.10, 1.86)	0.264	-0.007	0.923
	Energy	malate	0.56 (0.34, 0.91)	0.020	0.54 (0.20, 1.47)	0.228	0.003	0.786
	Energy	alpha-ketoglutarate	0.68 (0.48, 0.95)	0.026	0.71 (0.37, 1.37)	0.302	0.005	0.695
	Lipid	3-hydroxybutyrate (BHBA)	1.25 (1.07, 1.45)	0.004	1.25 (1.00, 1.56)	0.052	0.007	0.589
	Lipid	heptanoate (7:0)	0.45 (0.24, 0.85)	0.014	0.55 (0.20, 1.52)	0.249	0.018	0.167
	Lipid	taurochenodeoxycholate	0.78 (0.61, 1.00)	0.049	0.71 (0.50, 1.01)	0.057	0.016	0.229
	Lipid	palmitoylcarnitine	2.05 (1.20, 3.52)	0.009	1.92 (0.74, 5.00)	0.179	-0.021	0.324
	Lipid	hyodeoxycholate	1.24 (1.02, 1.51)	0.031	1.22 (0.91, 1.63)	0.177	0.003	0.774
	Lipid	palmitoleate (16:1n7)	1.63 (1.05, 2.54)	0.030	1.55 (0.71, 3.39)	0.275	-0.015	0.354
	Lipid	1-eicosatrienoylglycerophosphocholine	0.53 (0.34, 0.82)	0.004	0.69 (0.37, 1.29)	0.246	-0.028	0.073
	Lipid	1-oleoylglycerophosphocholine	0.49 (0.27, 0.88)	0.016	0.64 (0.21, 1.93)	0.431	-0.016	0.290
	Lipid	oleoylcarnitine	1.49 (1.10, 2.02)	0.011	1.29 (0.58, 2.86)	0.526	0.008	0.598
	Lipid	nonanoylcarnitine	1.48 (1.02, 2.15)	0.037	1.51 (1.07, 2.14)	0.02	0.014	0.384
	Lipid	***2-stearoylglycerophosphocholine***	***2.15 (1.48, 3.11)***	***<0.001***	***2.42 (1.18, 4.95)***	***0.016***	***-0.016***	***0.602***
	Lipid	2-tetradecenoyl carnitine	1.44 (1.08, 1.92)	0.013	1.62 (1.03, 2.55)	0.037	0.009	0.418
	Peptide	alpha-glutamyltyrosine	1.32 (1.02, 1.71)	0.032	1.12 (0.71, 1.76)	0.622	0.024	0.284
	Peptide	leucylleucine	0.61 (0.39, 0.94)	0.026	0.61 (0.32, 1.17)	0.139	-0.010	0.648
	Xenobiotics	theobromine	1.64 (1.06, 2.52)	0.026	2.08 (1.06, 4.07)	0.032	-0.051	0.466
	**Xenobiotics**	**thymol sulfate**	**1.32 (1.14, 1.53)**	**<0.001**	**1.36 (0.98, 1.88)**	**0.064**	**-0.034**	**0.457**

B2 (severity)								
	Amino_acid	2-hydroxyisobutyrate	1.47 (1.03, 2.09)	0.034	1.90 (1.13, 3.18)	0.015	0.013	0.142
	Amino_acid	indoleacetate	0.78 (0.65, 0.94)	0.011	0.74 (0.52, 1.07)	0.111	-0.003	0.558
	**Amino_acid**	**glutamate**	**1.39 (1.17, 1.65)**	**<0.001**	**1.36 (0.55, 3.34)**	**0.502**	**-0.025**	**0.828**
	Amino_acid	isobutyrylcarnitine	0.83 (0.72, 0.97)	0.017	0.84 (0.61, 1.14)	0.251	0.001	0.926
	CVs*	threonate	0.85 (0.73, 0.99)	0.039	0.80 (0.62, 1.03)	0.079	0.004	0.484
	Energy	phosphate	0.44 (0.23, 0.85)	0.014	0.35 (0.14, 0.86)	0.023	0.011	0.376
	Lipid	carnitine	0.65 (0.45, 0.93)	0.018	0.51 (0.27, 0.96)	0.036	0.002	0.576
	Lipid	palmitoylcarnitine	1.37 (1.03, 1.82)	0.030	1.51 (0.92, 2.50)	0.104	-0.004	0.722
	Lipid	***decanoylcarnitine***	***1.32 (1.17, 1.50)***	***<0.001***	***1.30 (1.01, 1.68)***	***0.043***	***0.006***	***0.359***
	**Lipid**	**1-heptadecanoylglycerophosphocholine**	**1.34 (1.18, 1.52)**	**<0.001**	**1.28 (0.86, 1.91)**	**0.219**	**-0.002**	**0.885**
	Lipid	epiandrosterone sulfate	0.86 (0.75, 0.99)	0.041	0.77 (0.65, 0.92)	0.003	0.014	0.075
	Lipid	1-arachidonoylglycerophosphoethanolamine	1.26 (1.01, 1.57)	0.042	1.10 (0.71, 1.69)	0.682	0.004	0.543
	Lipid	1-myristoylglycerophosphocholine	0.67 (0.50, 0.91)	0.009	0.69 (0.43, 1.13)	0.139	0.003	0.813
	Lipid	octadecanedioate	0.81 (0.69, 0.96)	0.014	0.88 (0.58, 1.33)	0.537	-0.009	0.434
	Lipid	taurolithocholate 3-sulfate	0.80 (0.70, 0.92)	0.002	0.83 (0.65, 1.05)	0.113	0.000	0.966
	Lipid	4-androsten-3beta,17beta-diol disulfate 2	1.23 (1.01, 1.49)	0.037	1.06 (0.77, 1.46)	0.735	0.002	0.811
	Nucleotide	inosine	1.09 (1.01, 1.18)	0.023	1.09 (0.99, 1.21)	0.090	-0.001	0.924
	Nucleotide	hypoxanthine	1.42 (1.08, 1.86)	0.012	1.23 (0.77, 1.96)	0.380	0.005	0.487
	Nucleotide	xanthine	0.67 (0.47, 0.96)	0.03	0.61 (0.31, 1.20)	0.154	-0.012	0.533
	Peptide	***gamma-glutamyltyrosine***	***1.74 (1.29, 2.35)***	***<0.001***	***1.70 (1.04, 2.77)***	***0.033***	***0.005***	***0.388***
	**Peptide**	**bradykinin, des-arg(9)**	**1.09 (1.05, 1.13)**	**<0.001**	**1.09 (1.00, 1.20)**	**0.056**	**0.000**	**0.949**
	Peptide	gamma-glutamylglutamate	1.13 (1.04, 1.24)	0.006	1.16 (0.92, 1.48)	0.215	-0.001	0.932
	Xenobiotics	salicylate	0.97 (0.96, 0.99)	0.009	0.97 (0.94, 1.00)	0.070	0.001	0.815
	Xenobiotics	***thymol sulfate***	***1.20 (1.10, 1.30)***	***<0.001***	***1.25 (1.05, 1.49)***	***0.014***	***0.002***	***0.922***
C2 (susceptibility)								
	Amino_acid	threonine	0.82 (0.72, 0.93)	0.003	0.76 (0.61, 0.95)	0.017	-0.004	0.396
	Amino_acid	N-acetylalanine	1.26 (1.03, 1.53)	0.024	1.38 (0.96, 1.99)	0.080	-0.038	0.072
	Amino_acid	glutamate	0.88 (0.81, 0.95)	0.001	0.90 (0.65, 1.25)	0.537	0.001	0.979
	Amino_acid	phenol sulfate	1.08 (1.01, 1.15)	0.021	1.04 (0.93, 1.16)	0.534	0.000	0.992
	CVs	threonate	0.92 (0.86, 0.98)	0.007	0.93 (0.84, 1.04)	0.224	0.001	0.638
	Lipid	linoleate (18:2n6)	1.25 (1.01, 1.55)	0.042	1.24 (0.95, 1.61)	0.107	-0.002	0.675
	Lipid	glycerophosphorylcholine (GPC)	0.95 (0.90, 0.99)	0.014	0.94 (0.86, 1.04)	0.237	0.000	0.817
	Lipid	dodecanedioate	0.89 (0.80, 1.00)	0.043	0.90 (0.78, 1.05)	0.179	0.007	0.380

Lipid	propionylcarnitine	0.90 (0.81, 0.99)	0.036	0.88 (0.74, 1.04)	0.139	0.001	0.497
Lipid	1-palmitoleoylglycerophosphocholine	1.20 (1.02, 1.40)	0.025	1.21 (0.97, 1.50)	0.084	-0.003	0.586
Lipid	decanoylcarnitine	1.05 (1.01, 1.09)	0.018	1.03 (0.93, 1.14)	0.549	0.001	0.642
Lipid	1-arachidonoylglycerophosphoethanolamine	1.16 (1.02, 1.32)	0.024	1.07 (0.90, 1.27)	0.445	0.002	0.437
Lipid	2-tetradecenoyl carnitine	1.06 (1.01, 1.11)	0.031	1.06 (0.96, 1.16)	0.231	0.002	0.360
Peptide	gamma-glutamyltyrosine	1.23 (1.08, 1.39)	0.001	1.27 (1.05, 1.53)	0.012	0.001	0.818
Peptide	glycylvaline	1.07 (1.01, 1.13)	0.026	1.05 (0.94, 1.17)	0.388	-0.002	0.827
Xenobiotics	quinate	0.96 (0.92, 1.00)	0.037	0.95 (0.87, 1.04)	0.239	0.002	0.723
Xenobiotics	saccharin	0.96 (0.94, 0.99)	0.001	0.96 (0.91, 1.00)	0.075	0.001	0.856
Xenobiotics	thymol sulfate	1.06 (1.00, 1.11)	0.046	1.07 (1.00, 1.15)	0.059	0.005	0.618

*: cofactors and vitamins. IVW, inverse variance weighted method with multiplicative random-effects. Associations that remained significant after multiple testing correction are in **boldface** type. Associations significant in sensitivity analysis ($P_{\text{weighted-median}}$ <0.05) are further in *italic* type.

Supplementary Table S5. Assessment of the causal relationship between T2D and metabolites (T2D as exposure).

Category	Metabolite	Nsnp	IVW Method		Weighted Median Method		MR-Egger Regression	
			Beta (95% CI)	*P*	Beta (95% CI)	*P*	Intercept	*P*
Amino_acid	histidine	82	-0.00(-0.01,0.00)	0.77	0.00(-0.01,0.01)	0.78	-0.001	0.048
**Amino_acid**	**threonine**	**81**	**0.01(0.00,0.02)**	**0.01**	0.01(-0.00,0.03)	0.08	-0.002	0.009
Amino_acid	N-acetylalanine	83	0.00(-0.00,0.01)	0.43	0.00(-0.00,0.01)	0.31	0.001	0.238
Amino_acid	***valine***	***83***	***0.01(0.00,0.01)***	***0.02***	***0.01(0.00,0.02)***	***0.04***	***0.000***	***0.526***
Amino_acid	2-hydroxyisobutyrate	83	0.00(-0.01,0.01)	0.74	0.00(-0.01,0.02)	0.74	-0.001	0.185
**Amino_acid**	**indoleacetate**	**82**	**0.02(0.00,0.03)**	**0.04**	0.01(-0.01,0.03)	0.55	0.002	0.207
Amino_acid	***glutamate***	***83***	***0.03(0.01,0.04)***	***0.00***	***0.03(0.01,0.05)***	***0.01***	***0.000***	***0.864***
Amino_acid	phenol sulfate	83	0.00(-0.02,0.02)	0.99	0.01(-0.02,0.05)	0.34	-0.001	0.562
.Amino_acid	isobutyrylcarnitine	83	-0.00(-0.02,0.01)	0.76	0.00(-0.02,0.02)	0.99	0.000	0.84
Amino_acid	hydroxytryptophan	83	0.00(-0.01,0.01)	0.45	0.00(-0.01,0.02)	0.60	0.002	0.011
Carbohydrate	***lactate***	***82***	***0.01(0.00,0.02)***	***0.03***	***0.02(0.01,0.04)***	***0.00***	***0.000***	***0.875***
Carbohydrate	***fructose***	***83***	***0.02(0.01,0.04)***	***0.00***	***0.04(0.01,0.06)***	***0.00***	***-0.001***	***0.509***
CVs*	threonate	83	-0.01(-0.03,0.01)	0.20	-0.00(-0.03,0.02)	0.69	0.001	0.594
Energy	malate	82	0.00(-0.01,0.02)	0.42	0.00(-0.01,0.02)	0.62	0.001	0.289
Energy	phosphate	83	-0.00(-0.01,0.00)	0.36	-0.00(-0.01,0.01)	0.72	0.000	0.444
Energy	alpha-ketoglutarate	82	0.01(-0.01,0.02)	0.39	0.01(-0.01,0.03)	0.36	0.001	0.351
Lipid	3-hydroxybutyrate (BHBA)	82	-0.00(-0.04,0.03)	0.81	-0.01(-0.05,0.04)	0.78	-0.001	0.67
Lipid	linoleate (18:2n6)	83	-0.00(-0.02,0.01)	0.39	-0.02(-0.03,0.00)	0.06	0.000	0.827
**Lipid**	**heptanoate (7:0)**	**83**	**-0.01(-0.02,-0.00)**	**0.00**	-0.01(-0.02,0.01)	0.35	0.001	0.442
Lipid	carnitine	81	0.00(-0.00,0.01)	0.57	0.00(-0.00,0.01)	0.37	0.000	0.745
Lipid	glycerophosphorylcholine (GPC)	83	-0.01(-0.02,0.00)	0.18	-0.01(-0.03,0.01)	0.41	-0.001	0.557
Lipid	taurochenodeoxycholate	83	0.02(-0.01,0.05)	0.15	0.03(-0.01,0.08)	0.17	0.003	0.278
Lipid	palmitoylcarnitine	83	0.01(-0.01,0.02)	0.35	0.00(-0.02,0.02)	0.85	0.002	0.13
Lipid	hyodeoxycholate	83	0.02(-0.01,0.04)	0.24	0.02(-0.03,0.06)	0.49	-0.002	0.521
Lipid	dodecanedioate	83	0.00(-0.01,0.02)	0.61	0.01(-0.01,0.04)	0.30	-0.001	0.341
Lipid	propionylcarnitine	81	0.01(-0.00,0.02)	0.22	0.01(-0.01,0.02)	0.49	0.001	0.089
Lipid	1-palmitoleoylglycerophosphocholine	83	-0.01(-0.02,0.01)	0.41	-0.00(-0.03,0.02)	0.68	0.001	0.457
Lipid	palmitoleate (16:1n7)	83	-0.01(-0.03,0.00)	0.08	-0.00(-0.03,0.02)	0.72	0.000	0.811
Lipid	1-eicosatrienoylglycerophosphocholine	83	-0.00(-0.02,0.01)	0.74	0.01(-0.01,0.03)	0.50	0.000	0.946
Lipid	decanoylcarnitine	83	-0.01(-0.03,0.01)	0.46	0.00(-0.02,0.03)	0.81	-0.002	0.225

Lipid	1-heptadecanoylglycerophosphocholine	83	-0.01(-0.03,0.01)	0.47	-0.00(-0.03,0.02)	0.74	-0.001	0.446
**Lipid**	**1-oleoylglycerophosphocholine**	**83**	**-0.01(-0.03,-0.00)**	**0.03**	-0.01(-0.03,0.00)	0.15	0.000	0.909
Lipid	epiandrosterone sulfate	82	0.02(-0.01,0.04)	0.20	-0.01(-0.05,0.03)	0.59	0.004	0.097
Lipid	oleoylcarnitine	82	0.00(-0.02,0.02)	0.96	-0.01(-0.03,0.02)	0.68	0.001	0.468
Lipid	1-arachidonoylglycerophosphoethanolamine	82	-0.01(-0.02,0.01)	0.33	0.00(-0.02,0.02)	0.83	-0.001	0.586
Lipid	nonanoylcarnitine	83	0.01(-0.00,0.03)	0.14	0.03(-0.00,0.06)	0.07	-0.002	0.339
Lipid	2-stearoylglycerophosphocholine	82	-0.01(-0.03,0.01)	0.22	-0.01(-0.03,0.01)	0.38	0.000	0.929
Lipid	2-tetradecenoyl carnitine	83	-0.01(-0.03,0.02)	0.59	-0.01(-0.04,0.02)	0.70	0.002	0.387
Lipid	1-myristoylglycerophosphocholine	82	-0.01(-0.02,0.01)	0.50	0.01(-0.02,0.03)	0.50	0.000	0.745
Lipid	octadecanedioate	83	-0.00(-0.02,0.01)	0.90	-0.00(-0.03,0.02)	0.88	0.000	0.852
Lipid	taurolithocholate 3-sulfate	81	0.00(-0.02,0.03)	0.79	-0.00(-0.05,0.04)	0.84	0.004	0.121
Lipid	4-androsten-3beta,17beta-diol disulfate 2	83	0.01(-0.01,0.03)	0.25	-0.01(-0.03,0.02)	0.63	0.003	0.051
**Nucleotide**	**inosine**	**83**	**-0.05(-0.09,-0.01)**	**0.01**	-0.06(-0.12,0.01)	0.08	0.003	0.369
Nucleotide	hypoxanthine	83	0.00(-0.01,0.02)	0.60	0.00(-0.01,0.02)	0.64	0.000	0.701
Nucleotide	xanthine	83	-0.00(-0.01,0.01)	0.81	-0.01(-0.02,0.01)	0.59	0.000	0.778
Peptide	***gamma-glutamyltyrosine***	***83***	***0.02(0.01,0.02)***	***0.00***	***0.02(0.01,0.03)***	***0.00***	***0.001***	***0.145***
Peptide	glycylvaline	82	0.02(-0.02,0.05)	0.30	0.03(-0.02,0.08)	0.26	0.000	0.866
Peptide	bradykinin, des-arg(9)	82	-0.00(-0.04,0.04)	0.90	-0.02(-0.08,0.04)	0.56	0.003	0.354
Peptide	alpha-glutamyltyrosine	79	-0.01(-0.03,0.01)	0.46	0.00(-0.04,0.04)	0.93	-0.002	0.393
Peptide	gamma-glutamylglutamate	81	0.02(-0.01,0.05)	0.17	0.01(-0.04,0.05)	0.78	0.001	0.647
Peptide	leucylleucine	83	-0.00(-0.02,0.01)	0.80	-0.01(-0.04,0.02)	0.52	-0.001	0.579
Xenobiotics	salicylate	82	0.01(-0.06,0.08)	0.80	0.05(-0.05,0.16)	0.33	-0.011	0.081
Xenobiotics	quinate	83	0.02(-0.01,0.06)	0.19	0.03(-0.02,0.08)	0.28	0.002	0.56
Xenobiotics	theobromine	83	-0.00(-0.03,0.02)	0.80	-0.00(-0.04,0.04)	0.98	-0.001	0.687
Xenobiotics	saccharin	80	-0.00(-0.05,0.05)	0.94	-0.02(-0.09,0.05)	0.52	0.001	0.816
Xenobiotics	thymol sulfate	83	-0.01(-0.06,0.03)	0.50	-0.01(-0.08,0.07)	0.81	0.001	0.89

*: cofactors and vitamins. Nsnp: number of valid genetic instruments after Steiger filtering. IVW, inverse variance weighted method with multiplicative random-effects. Associations of nominal significance in primary analysis (P_{IVW} <0.05) are in **boldface** type. Associations that remained significant in sensitivity analysis ($P_{weighted\text{-}median}$ <0.05) are further in *italic* type.

Supplementary Table S6. Direct effects of T2D-associated metabolites on COVID-19 phenotypes conditioning on T2D.

COVID-19 Outcome	Category	Metabolites	Nsnp	MVMR IVW Method	
				OR (95% CI)	*P*
A2					
	Amino_acid	indoleacetate	4	1.00(0.46,2.16)	0.995
	Amino_acid	valine	2	2.88(0.23,35.32)	0.408
	Carbohydrate	fructose	2	0.45(0.19,1.04)	0.063
	Carbohydrate	lactate	6	0.76(0.28,2.07)	0.597
	Lipid	heptanoate (7:0)	12	0.74(0.28,1.95)	0.540
B2					
	Amino_acid	glutamate	2	1.15(0.64,2.07)	0.641
	Amino_acid	indoleacetate	4	0.83(0.48,1.42)	0.489
	Nucleotide	inosine	3	1.08(0.96,1.21)	0.186
	**Peptide**	**gamma-glutamyltyrosine**	**14**	**2.15(1.13,4.09)**	**0.020**
C2					
	**Amino_acid**	**glutamate**	**2**	**0.73(0.56,0.96)**	**0.025**
	Peptide	gamma-glutamyltyrosine	14	1.26(0.93,1.71)	0.142

Associations of nominal significance in primary analysis ($P_{MV\text{-}IVW}$ <0.05) are in **boldface** type.

Supplementary Table S7. Assessment of the causal relationship between BMI and metabolites (BMI as exposure).

Category	Metabolite	Nsnp	IVW Method		Weighted Median Method		MR-Egger Regression	
			Beta (95% CI)	*P*	Beta (95% CI)	*P*	Intercept	*P*
Amino_acid	histidine	67	0.00 (-0.01, 0.01)	0.624	0.00 (-0.01, 0.02)	0.676	0.000	0.380
Amino_acid	threonine	66	-0.01 (-0.04, 0.01)	0.276	-0.01 (-0.04, 0.03)	0.744	0.000	0.748
Amino_acid	N-acetylalanine	67	0.01 (-0.01, 0.02)	0.455	0.01 (-0.01, 0.03)	0.418	-0.001	0.484
**Amino_acid**	**valine**	**67**	**0.01 (0.00, 0.03)**	**0.015**	**0.01 (-0.01, 0.03)**	**0.331**	**0.001**	**0.137**
Amino_acid	2-hydroxyisobutyrate	66	0.00 (-0.03, 0.02)	0.917	-0.01 (-0.05, 0.03)	0.540	0.002	0.093
Amino_acid	indoleacetate	66	0.01 (-0.02, 0.04)	0.477	0.02 (-0.03, 0.07)	0.478	-0.001	0.263
**Amino_acid**	**glutamate**	**67**	**0.05 (0.02, 0.09)**	**0.005**	**0.04 (-0.01, 0.10)**	**0.088**	**0.001**	**0.576**
**Amino_acid**	**phenol sulfate**	**67**	**-0.06 (-0.11, -0.01)**	**0.017**	**-0.03 (-0.11, 0.04)**	**0.402**	**0.001**	**0.586**
Amino_acid	isobutyrylcarnitine	67	0.02 (-0.01, 0.06)	0.207	0.03 (-0.03, 0.08)	0.358	-0.001	0.504
Amino_acid	hydroxytryptophan	67	0.01 (-0.01, 0.03)	0.224	0.01 (-0.02, 0.04)	0.455	0.001	0.513
Carbohydrate	lactate	67	0.02 (-0.01, 0.04)	0.153	0.00 (-0.03, 0.04)	0.786	0.001	0.318
Carbohydrate	fructose	67	0.01 (-0.02, 0.05)	0.468	0.01 (-0.05, 0.07)	0.737	-0.001	0.440
CVs*	threonate	67	0.00 (-0.03, 0.03)	0.896	0.01 (-0.04, 0.07)	0.702	-0.001	0.394
Energy	malate	67	0.00 (-0.03, 0.02)	0.952	0.01 (-0.03, 0.05)	0.564	0.001	0.505
Energy	phosphate	67	-0.01 (-0.02, 0.00)	0.150	-0.01 (-0.03, 0.01)	0.287	0.000	0.907
Energy	alpha-ketoglutarate	67	0.02 (-0.02, 0.05)	0.385	0.02 (-0.03, 0.08)	0.428	0.000	0.920
Lipid	3-hydroxybutyrate (BHBA)	66	-0.02 (-0.1, 0.06)	0.574	-0.04 (-0.14, 0.07)	0.508	-0.001	0.767
Lipid	linoleate (18:2n6)	67	0.00 (-0.03, 0.02)	0.763	-0.01 (-0.05, 0.02)	0.449	0.000	0.881
**Lipid**	**heptanoate (7:0)**	**66**	**-0.03 (-0.05, -0.01)**	**0.001**	**-0.03 (-0.05, 0.00)**	**0.096**	**0.001**	**0.542**
Lipid	carnitine	67	0.01 (0.00, 0.02)	0.077	0.01 (-0.01, 0.02)	0.552	0.001	0.014
Lipid	glycerophosphorylcholine (GPC)	67	-0.02 (-0.05, 0.01)	0.195	-0.03 (-0.08, 0.02)	0.205	0.000	0.876
Lipid	***taurochenodeoxycholate***	***66***	***0.08 (0.02, 0.14)***	***0.012***	***0.10 (0.00, 0.20)***	***0.044***	***0.000***	***0.891***
Lipid	palmitoylcarnitine	67	-0.02 (-0.05, 0.01)	0.190	0.00 (-0.05, 0.05)	0.924	0.001	0.472
Lipid	hyodeoxycholate	66	0.03 (-0.04, 0.10)	0.412	0.04 (-0.07, 0.15)	0.486	-0.001	0.761
Lipid	dodecanedioate	67	-0.02 (-0.06, 0.02)	0.224	-0.02 (-0.08, 0.04)	0.430	0.001	0.443
**Lipid**	**propionylcarnitine**	**66**	**0.03 (0.00, 0.05)**	**0.032**	**0.03 (0.00, 0.06)**	**0.071**	**-0.001**	**0.427**
Lipid	1-palmitoleoylglycerophosphocholine	67	-0.02 (-0.06, 0.01)	0.232	-0.02 (-0.07, 0.03)	0.501	-0.001	0.679
Lipid	palmitoleate (16:1n7)	66	0.01 (-0.03, 0.05)	0.617	0.01 (-0.04, 0.07)	0.694	0.000	0.842
Lipid	1-eicosatrienoylglycerophosphocholine	67	-0.02 (-0.06, 0.01)	0.234	-0.03 (-0.08, 0.02)	0.287	0.001	0.472
Lipid	decanoylcarnitine	65	-0.03 (-0.07, 0.01)	0.172	-0.01 (-0.07, 0.05)	0.821	-0.003	0.086

Lipid	1-heptadecanoylglycerophosphocholine	67	-0.04 (-0.08, 0.00)	0.071	0.00 (-0.06, 0.06)	0.956	-0.002	0.156
**Lipid**	**1-oleoylglycerophosphocholine**	**66**	**-0.03 (-0.06, -0.01)**	**0.014**	**-0.03 (-0.07, 0.01)**	**0.149**	**-0.002**	**0.151**
Lipid	epiandrosterone sulfate	67	-0.02 (-0.07, 0.03)	0.461	-0.02 (-0.11, 0.07)	0.654	0.001	0.645
Lipid	oleoylcarnitine	67	-0.02 (-0.06, 0.02)	0.251	-0.01 (-0.07, 0.04)	0.647	0.000	0.874
Lipid	1-arachidonoylglycerophosphoethanolamine	67	-0.02 (-0.05, 0.00)	0.087	-0.02 (-0.05, 0.02)	0.455	0.001	0.220
Lipid	nonanoylcarnitine	67	-0.01 (-0.06, 0.04)	0.697	-0.01 (-0.08, 0.06)	0.878	-0.002	0.317
**Lipid**	**2-stearoylglycerophosphocholine**	**67**	**-0.04 (-0.07, 0.00)**	**0.028**	**-0.03 (-0.08, 0.03)**	**0.298**	**-0.001**	**0.330**
**Lipid**	**2-tetradecenoyl carnitine**	**67**	**-0.05 (-0.09, 0.00)**	**0.042**	**-0.02 (-0.09, 0.04)**	**0.486**	**-0.001**	**0.718**
Lipid	1-myristoylglycerophosphocholine	67	-0.03 (-0.06, 0.01)	0.122	-0.02 (-0.07, 0.03)	0.455	0.000	0.874
Lipid	octadecanedioate	67	-0.02 (-0.05, 0.02)	0.281	-0.01 (-0.06, 0.05)	0.852	-0.001	0.628
Lipid	taurolithocholate 3-sulfate	67	0.01 (-0.06, 0.08)	0.799	0.04 (-0.05, 0.14)	0.373	-0.006	0.049
Lipid	4-androsten-3beta,17beta-diol disulfate 2	67	-0.01 (-0.05, 0.04)	0.801	0.00 (-0.07, 0.06)	0.956	0.001	0.484
Nucleotide	inosine	67	-0.02 (-0.13, 0.1)	0.782	-0.03 (-0.19, 0.14)	0.745	-0.003	0.495
Nucleotide	hypoxanthine	67	0.00 (-0.03, 0.02)	0.715	0.02 (-0.02, 0.05)	0.380	0.000	0.717
Nucleotide	***xanthine***	***67***	***0.04 (0.02, 0.06)***	***0.001***	***0.05 (0.00, 0.09)***	***0.035***	***-0.001***	***0.540***
Peptide	***gamma-glutamyltyrosine***	***67***	***0.04 (0.02, 0.06)***	***<0.001***	***0.05 (0.02, 0.08)***	***0.003***	***0.000***	***0.701***
Peptide	glycylvaline	66	0.07 (0.00, 0.14)	0.062	0.08 (-0.03, 0.20)	0.152	-0.001	0.718
**Peptide**	**bradykinin, des-arg(9)**	**67**	**0.11 (0.00, 0.21)**	**0.043**	**0.07 (-0.08, 0.21)**	**0.374**	**0.000**	**0.940**
**Peptide**	**alpha-glutamyltyrosine**	**67**	**-0.06 (-0.11, -0.01)**	**0.016**	**-0.03 (-0.12, 0.05)**	**0.486**	**0.000**	**0.960**
Peptide	gamma-glutamylglutamate	65	-0.05 (-0.11, 0.01)	0.092	0.00 (-0.10, 0.10)	0.974	-0.002	0.407
Peptide	leucylleucine	67	0.02 (-0.03, 0.06)	0.393	0.03 (-0.04, 0.10)	0.355	-0.001	0.686
Xenobiotics	salicylate	67	-0.08 (-0.22, 0.06)	0.278	-0.14 (-0.38, 0.11)	0.277	0.014	0.060
**Xenobiotics**	**quinate**	**67**	**0.12 (0.05, 0.20)**	**0.002**	**0.04 (-0.09, 0.16)**	**0.580**	**0.000**	**0.960**
Xenobiotics	theobromine	67	0.00 (-0.07, 0.06)	0.968	0.05 (-0.04, 0.15)	0.298	0.005	0.091
Xenobiotics	saccharin	67	-0.03 (-0.14, 0.08)	0.561	-0.01 (-0.18, 0.15)	0.872	-0.003	0.596
Xenobiotics	thymol sulfate	67	-0.02 (-0.13, 0.09)	0.755	-0.07 (-0.23, 0.10)	0.444	-0.001	0.788

*: cofactors and vitamins. Nsnp: number of valid genetic instruments after Steiger filtering. IVW, inverse variance weighted method with multiplicative random-effects. Associations of nominal significance in primary analysis (P_{IVW} <0.05) are in **boldface** type. Associations that remained significant in sensitivity analysis ($P_{\text{weighted-median}}$ <0.05) are further in *italic* type.

Supplementary Table S8. Direct effects of BMI-associated metabolites on COVID-19 phenotypes conditioning on BMI.

COVID-19 Outcome	Category	Metabolites	Nsnp	MVMR IVW Method	
				OR (95% CI)	*P*
A2					
	**Amino acid**	**valine**	**4**	**8.89(1.22,64.64)**	**0.031**
	Lipid	1-oleoylglycerophosphocholine	10	0.72(0.36,1.46)	0.364
	Lipid	2-stearoylglycerophosphocholine	3	0.96(0.46,2.00)	0.921
	Lipid	2-tetradecenoyl carnitine	12	1.22(0.84,1.75)	0.295
	Lipid	heptanoate (7:0)	24	0.73(0.36,1.48)	0.379
	Lipid	taurochenodeoxycholate	7	0.93(0.69,1.24)	0.625
	**Peptide**	**alpha-glutamyltyrosine**	**10**	**1.40(1.02,1.94)**	**0.039**
B2					
	Amino acid	glutamate	3	1.14(0.71,1.82)	0.596
	Nucleotide	xanthine	3	0.88(0.50,1.55)	0.655
	Peptide	bradykinin, des-arg(9)	11	1.07(0.99,1.16)	0.106
	**Peptide**	**gamma-glutamyltyrosine**	**26**	**1.62(1.03,2.54)**	**0.035**
C2					
	**Amino acid**	**glutamate**	**3**	**0.82(0.69,0.98)**	**0.031**
	Amino acid	phenol sulfate	7	1.00(0.91,1.10)	0.95
	Lipid	2-tetradecenoyl carnitine	12	1.05(0.96,1.15)	0.294
	Lipid	propionylcarnitine	21	0.95(0.83,1.09)	0.47
	Peptide	gamma-glutamyltyrosine	26	1.16(0.98,1.39)	0.089
	**Peptide**	**alpha-glutamyltyrosine**	**10**	**1.10(1.02,1.19)**	**0.012**
	Xenobiotics	quinate	4	1.00(0.93,1.07)	0.947

Associations of nominal significance in primary analysis ($P_{\text{MV-IVW}}$ <0.05) are in **boldface** type.

Supplementary Table S9. Association between metabolites and COVID-19 phenotypes after adjusting for other metabolites with correlated serum levels.

COVID-19 Outcome	Metabolite_ID	Metabolite	Pearson's Correlation (*r*)	MV-IVW Method		
				Beta	SE	*P*
A2	M37203	4-androsten-3beta,17beta-diol disulfate 2	0.63	0.275	0.188	0.145
	M33973	epiandrosterone sulfate		-0.181	0.121	0.133
B2	M37203	4-androsten-3beta,17beta-diol disulfate 2	0.63	0.298	0.107	0.005
	**M33973**	**epiandrosterone sulfate**		**-0.206**	**0.067**	**0.002**
C2	M37203	4-androsten-3beta,17beta-diol disulfate 2	0.63	0.078	0.052	0.130
	M33973	epiandrosterone sulfate		-0.018	0.033	0.577
A2	**M35255**	**2-stearoylglycerophosphocholine**	0.75	**1.980**	**0.474**	**0.000**
	M33960	1-oleoylglycerophosphocholine		-2.349	0.611	0.000
B2	M32322	glutamate	0.35	0.264	0.271	0.330
	**M02734**	**gamma-glutamyltyrosine**		**0.477**	**0.174**	**0.006**
B2	M32322	glutamate	0.61	0.224	0.184	0.223
	M36738	gamma-glutamylglutamate		0.105	0.048	0.031
B2	**M02734**	**gamma-glutamyltyrosine**	0.32	**0.529**	**0.147**	**0.000**
	M36738	gamma-glutamylglutamate		0.089	0.070	0.204
B2	M03127	hypoxanthine	0.28	0.833	0.225	0.000
	**M32739**	**xanthine**		**-1.074**	**0.303**	**0.000**
C2	**M02734**	**gamma-glutamyltyrosine**	0.35	**0.220**	**0.074**	**0.003**
	M32322	glutamate		-0.059	0.114	0.604
C2	M32452	propionylcarnitine	0.37	-0.124	0.059	0.035
	**M02734**	**gamma-glutamyltyrosine**		**0.208**	**0.069**	**0.002**

Associations of nominal significance in primary analysis ($P_{\text{MV-IVW}}$ <0.05) are in **boldface** type. No correlated metabolites were found for alpha-glutamyltyrosine.
